# Ectopic expression of a truncated NLR gene from wild *Arachis* enhances resistance to *Fusarium oxysporum*

**DOI:** 10.3389/fpls.2024.1486820

**Published:** 2024-11-13

**Authors:** Amanda Cristina de Araújo, Ana Cristina Miranda Brasileiro, Andressa da Cunha Quintana Martins, Priscila Grynberg, Roberto Coiti Togawa, Mario Alfredo de Passos Saraiva, Robert Neil Gerard Miller, Patricia Messenberg Guimaraes

**Affiliations:** ^1^ Departamento de Biologia Celular, Universidade de Brasília, Brasília, DF, Brazil; ^2^ Embrapa Recursos Genéticos e Biotecnologia, Parque Estação Biológica – PqEB, Brasília, DF, Brazil; ^3^ National Institute of Science and Technology - INCT PlantStress Biotech, EMBRAPA, Brasilia, DF, Brazil

**Keywords:** truncated-NLR, transcriptome, plant defense, stress, fungi

## Abstract

*Fusarium oxysporum* causes devastating vascular wilt diseases in numerous crop species, resulting in substantial yield losses. The *Arabidopsis thaliana*-*F. oxysporum f.sp. conglutinans* (FOC) model system enables the identification of meaningful genotype–phenotype correlations and was applied in this study to evaluate the effects of overexpressing an NLR gene (*AsTIR19*) from *Arachis stenosperma* against pathogen infection. *AsTIR19* overexpression (OE) lines exhibited enhanced resistance to FOC without any discernible phenotype penalties. To elucidate the underlying resistance mechanisms mediated by *AsTIR19* overexpression, we conducted whole transcriptome sequencing of an AsTIR19-OE line and non-transgenic wild-type (WT) plants inoculated and non-inoculated with FOC using Illumina HiSeq4000. Comparative analysis revealed 778 differentially expressed genes (DEGs) attributed to transgene overexpression, while fungal inoculation induced 434 DEGs in the OE line, with many falling into defense-related Gene Ontology (GO) categories. GO and KEGG enrichment analysis showed that DEGs were enriched in the phenylpropanoid and flavonoid pathways in the OE plants. This comprehensive transcriptomic analysis underscores how *AsTIR19* overexpression reprograms transcriptional networks, modulating the expression of stress-responsive genes across diverse metabolic pathways. These findings provide valuable insights into the molecular mechanisms underlying the role of this NLR gene under stress conditions, highlighting its potential to enhance resistance to *Fusarium oxysporum*.

## 1 Introduction

While advances in biotechnology, genetics and breeding have resulted in significant improvements in crop productivity (FAO et al., 2023), various abiotic or biotic stresses, such as those caused by pathogens, insects, soil salinity, and drought still pose enormous threats to agricultural production. Furthermore, the interplay of various stresses due to the multifactorial nature of climate change poses an even greater threat to major crops, global food production, and food security (Rivero et al., 2022).

Wild plant species possess a wide range of valuable agronomical traits, such as resilience to both biotic and abiotic stresses, which are relevant for introgression into contemporary crop varieties for adaptation to changing global environmental conditions (Bohra et al., 2022). The identification of disease resistance genes from wild species or closely related varieties of target crops presents, therefore, a promising strategy for genetic enhancement. Wild *Arachis* species have gained significant attention as sources of resistance to various pathogens, including fungi, nematodes, and insects (Sharma et al., 2003; Guimarães et al., 2012; Mota et al., 2018), as well as tolerance to hydric stress (Vinson et al., 2020; Brasileiro et al., 2021; Puli et al., 2021). These species serve as valuable resources for plant improvement, with alleles from wild *Arachis* now introgressed into cultivated peanut (Fonceka et al., 2012; Dutra et al., 2018; Bertioli et al., 2021; Moretzsohn et al., 2023) and transferred to different plant species through transgenic approaches (Mota et al., 2019; Brasileiro et al., 2021; Guimaraes et al., 2022; Da Silva Ferreira et al., 2024).

NLR (Nucleotide-binding site and Leucine-rich repeat) genes form a large family of resistance genes (*R*) in plants and are key components of the plant immune system, detecting pathogens and triggering defense responses (Dangl and Jones, 2001; Lu and Tsuda, 2021). NLR proteins act as intracellular receptors that recognize pathogen effectors through mechanisms that can involve direct binding, guards, and decoy strategies (Cesari, 2018). Their activation prompts Effector-Triggered Immunity (ETI), which is often a result of a gene-for-gene relationship with their cognate effectors that results in a localized hypersensitivity response (HR) at the site of infection (Pottinger and Innes, 2020). Plant NLRs typically display a central nucleotide-binding site (NBS) domain, an N-terminal region, which can contain a toll/interleukin-1 receptor (TIR) domain (TNLs) or a coiled-coil (CC) domain (CNLs), together with a variable C-terminal leucine-rich repeat (LRR) domain. In addition, atypical truncated NLRs lacking the LRR domain, namely TIR-NBS (TN) and CC-NBS (CN), are also found in higher plant genomes. Although taxonomically specific functionality might restrict the application of *R* genes across distinct plant groups (Jones et al., 2016), successful transfer of NLRs between distant species has been observed (Zhao and Fernald, 2005; Zhang et al., 2017; Xu et al., 2018). The application of truncated NLRs in enhancing immunity against fungal and bacterial pathogens has been successfully explored in *Arabidopsis* and tobacco (Staal et al., 2008; Wang et al., 2013; Zhao et al., 2015; Roth et al., 2017; Barragan et al., 2021; Son et al., 2021; Guimaraes et al., 2022). While overexpression of NLRs often results in autoimmunity and significant fitness costs, different mechanisms controlling transcript levels of truncated, helper, and inhibitor NLRs make them potentially engineerable to enhance plant defenses while mitigating their fitness costs (Lai and Eulgem, 2018).

*Fusarium oxysporum* is a common soil fungal pathogen causing vascular wilt in a wide variety of plants, leading to symptoms such as yellowing, defoliation, and plant death. This fungus can infect over 100 different host species, causing significant losses in crops such as melon, tomato, cotton, and banana (Dean et al., 2012). Recent research has advanced our understanding of the genetics of this fungus, providing molecular insights into the mechanisms responsible for virulence factors (Rauwane et al., 2020; Zuriegat et al., 2021), host adaptation (Jangir et al., 2021), pathogenicity chromosomes (Ma et al., 2010; Li et al., 2020), and the complexity of the species (Li et al., 2020). Control measures against the pathogen have included cultural, biological, and chemical strategies. While these approaches may reduce disease incidence, they do not eliminate the soilborne pathogen and can be onerous, negatively affecting the environment (Li et al., 2020). As such, the employment of resistant/tolerant varieties is important for more effective and sustainable integrated pathogen management. Resistance to *F. oxysporum* is observed in various crop species and is often mediated by ETI immune responses (Beckman and Roberts, 1995). Nonetheless, *F. oxysporum* has frequently shown to be able to overcome such defenses through its ability to rapidly adapt and evolve, posing significant challenges to long-term resistance in crops (Michielse and Rep, 2009). Thus, identifying and integrating new sources of resistance, including those from wild relatives, and employing advanced biotechnological approaches, such as gene pyramiding and gene editing, are imperative for developing durable resistance.

Ectopic expression of defense genes in transgenic plants, such as chitinases, pathogenesis-related proteins (PRs), defensins, thaumatin-like proteins and transcription factors (TFs) in transgenic plants, has shown success in reducing infection by *F. oxysporum* in different plant species (Swathi Anuradha et al., 2008; Mahdavi et al., 2012; Liu et al., 2016; Diao et al., 2022). Likewise, host-induced gene silencing (HIGS) through RNA interference (RNAi) targeting genes essential for the pathogen virulence or development has rendered enhanced tolerance to the pathogen (Ghag et al., 2014; Singh et al., 2020; Fernandes et al., 2016; Hu et al., 2016; Bharti et al., 2017). Wild species have been effectively utilized in breeding programs to enhance resistance against *F. oxysporum* in crops such as tomato, passion fruit, and eggplant (Melo et al., 2020; Chitwood-Brown et al., 2021; Tassone et al., 2022). Additionally, the overexpression of an NLR from the wild diploid banana *Musa acuminata* in transgenic plants conferred resistance to the devastating *F. oxysporum f.sp. cubense* race 4 (Dale et al., 2017).

Recently, we demonstrated that overexpressing *AsTIR19*, a truncated NLR (TNx) gene from the wild species *Arachis stenosperma*, enhanced resistance against the necrotrophic plant pathogenic fungus *Sclerotinia sclerotiorum* in tobacco plants (Guimaraes et al., 2022). In this study, we investigated the effects of *AsTIR19* overexpression on reducing the infection of the hemibiotrophic *F. oxysporum f.sp. conglutinans* (FOC) in *Arabidopsis* plants. We also analyzed the transcriptome reprogramming in a selected AsTIR-OE line due to *AsTIR19* overexpression inoculated and non-inoculated with the pathogen. The enrichment of stress–responsive genes involved in defense metabolic pathways in the transgenic OE line, such as the phenylpropanoid and flavonoid pathways, combined with the subsequent upregulation of genes in the salicylic acid (SA) and jasmonic acid-ethylene (JA-ET) signaling pathways following FOC inoculation, indicate a coordinated and robust response against the pathogen invasion.

While leveraging NLR genes is crucial for enhancing crop resilience, a deeper understanding of the molecular mechanisms behind the improved resistance or tolerance provided by their ectopic expression is essential to minimize unintended effects, and support the development of more effective genetic strategies for crop improvement.

## 2 Materials and methods

### 2.1 Plant transformation

*Arabidopsis thaliana* (ecotype Col-0) plants were transformed using the floral dip method (Clough and Bent, 1998) with *Agrobacterium tumefaciens* strain ‘GV3101’ harboring the 1,920 bp coding sequence of *AsTIR19* from *A. stenosperma* cloned in the binary vector pPZP-BAR (Guimaraes et al., 2022). Transformed plants were maintained in a controlled growth chamber (21°C with a 12 h photoperiod and light intensity of 200 μmols.m^−2^.s^−1^) and T0 glufosinate-resistant and eGFP-positive transformants were selected to produce the T1 generation. Transformants were screened repeatedly for glufosinate ammonium herbicide resistance to obtain homozygous *AsTIR19* overexpressing (OE) lines, at the T2 generation, as described previously (Vinson et al., 2020). The transgenic status of OE-lines was confirmed by the presence of the selection marker gene *bar* in glufosinate-resistant seedlings at T1 generation by PCR analysis, with the relative expression of the *AsTIR19* transgene further determined in OE lines at T2 generation by qRT-PCR analysis, as described below, using specific primers (**Supplementary Table S1**). To assess the germination rate of the AsTIR19-OE seeds, T2 seeds were sown in 80 mL pots containing soil substrate (Carolina Soil, CSC, Brazil) and monitored daily for germination over a 12-day period. The germination rate was determined in relation to total sown seeds, in each OE line.

### 2.2 AsTIR19 OE lines and FOC bioassays

Seeds from wild-type (WT) plants and AsTIR19-OE T2 lines were ethanol-disinfected, sown in 200 mL pots containing sterilized substrate (Carolina Soil, CSC, Brazil) and maintained in a growth chamber as described above. *F. oxysporum* f.sp*. conglutinans* PHW 699-3 (ATCC 58110) (FOC) (Hou et al., 2014) was grown on a plate containing Potato Dextrose Agar (PDA) medium and incubated for two days at 28°C, then transferred to a liquid Potato Broth Culture (PBC) and incubated for additional three days on a rotary shaker (110 rpm) at 28°C. The culture was filtered using sterilized filter paper, centrifuged, and diluted to 10^6^ conidiospores/mL in sterile water.

Four-week-old plants (AsTIR19-OE and WT) were inoculated at four equidistant points per individual on the substrate with 5 mL of the diluted FOC suspension. The non-inoculated control plants underwent the same treatment but were inoculated only with autoclaved distilled water. Following FOC inoculation, plants were maintained in a growth chamber (light/dark cycle of 8/16 h, 23°C) and arranged in a randomized design. Assessment of FOC infection was conducted at 7 and 14 days after inoculation (DAI) using the disease index (DI) according to the scale proposed by Diener and Ausubel (2005). The symptoms score applied was as follows: 0- plants are dead; 1- older leaves are dead and young leaves severely stunted; 2- older leaves are chlorotic, yellow, or dead and younger leaves are stunted; 3- older leaves have vascular chlorosis and the rosette appears compact because leaves are stunted; 4- leaf petioles are stunted; and 5- plants are indistinguishable from non-inoculated plants. Three biological replicates of five plants from each OE line and WT were evaluated at three time points (0, 7, 14 DAI) for symptom development. A mean DI was used to conduct a Tukey test to evaluate the significance of the results.

### 2.3 RNA extraction and sequencing

Total RNA was extracted from three pools of root tissues from five T2 seedlings of AsTIR19-OE-11 line and WT plants, submitted to FOC inoculation (7 DAI) and corresponding non-inoculated controls. Extraction was performed using the TRIzol^®^ Reagent (Ambion^®^, Foster City, CA, USA), and purified using the RNeasy Plant Mini Kit (Qiagen, Hilden, Germany). RNA integrity and quantity were verified using gel electrophoresis and a 2100 Bioanalyzer system (Agilent Technologies, Santa Clara, CA, USA). Twelve cDNA libraries, consisting of triplicates of AsTIR19-inoculated (OE-11-I), AsTIR19-non-inoculated (OE-11-NI), Wild-type-inoculated (WT-I) and Wild-type-non-inoculated (WT-NI) were prepared with the “TruSeq Stranded mRNA Library Prep Kit” (Illumina Inc., San Diego, CA, USA) and sequenced on an Illumina HiSeq-4000 at Macrogen Inc. (Seoul, South Korea). Transcriptomic raw data is available in the Sequence Read Archive (NCBI-SRA) repository under the Bioproject ID PRJNA1125443.

### 2.4 *In silico* expression profiling

The *A. thaliana* reference genome (TAIR10; https://www.arabidopsis.org/) was used for analysis of the sequence data. Sequence analysis and processing were conducted using FASTp (Chen et al., 2018), STAR (Dobin and Gingeras, 2016), and HTSeq (Anders et al., 2015). Preprocessed data were statistically validated using ClustVis (Metsalu and Vilo, 2015). Differential gene expression analysis was performed with the edgeR package (Robinson et al., 2010).

Genes were classified as differentially expressed (DEGs) if the adjusted p-value was <0.05, as determined by the False Discovery Rate (FDR) method following the Benjamini-Hochberg procedure (Haynes, 2013), and exhibited a minimum 4-fold change in expression (log2FC > 2 or < -2) between samples. DEGs were identified by comparing the following conditions: (i) OE-11-NI vs. WT-NI, (ii) OE-11-I vs. OE-11-NI, (iii) WT-I vs. WT-NI, and (iv) OE-11-I vs. WT-I.

Volcano plots of DEGs were graphically represented using the R package ggplot2 (Wickham, 2016) and BioRender (BioRender.com). To analyze the overlap of DEGs between samples, a Venn diagram was created using the InteractiVenn tool (www.interactivenn.net; Heberle et al., 2015).

A cluster heatmap was generated using ClustVis (https://biit.cs.ut.ee/clustvis/) to visualize the expression profiles of differentially expressed genes (DEGs). All genes identified as DEGs in at least one category were included to compare gene expression patterns across samples. The average correlation was used as the clustering distance metric for rows, while the average Euclidean distance was used for clustering columns.

Manhattan plots were used to summarize the positions and frequencies of DEGs across the five *A. thaliana* chromosomes. Each identified DEG was mapped to its specific genomic location (TAIR10; https://www.arabidopsis.org/). The p-values were transformed using the negative logarithm base 10 (-log10) to enhance the visualization of statistical significance, with higher -log10 (p-values) indicating greater significance. To account for multiple testing, the Bonferroni correction was applied, setting a significance threshold of p < 0.00001. Graphical representation was created using the R package qqman (Turner, 2014).

### 2.5 Functional analysis of DEGs

To identify significant associations between gene sets and ontological annotations, we employed the hypergeometric test available in the FUNC package (Prüfer et al., 2007). This test was used to assess the overrepresentation of GO terms among the DEGs, following established procedures (Vinson et al., 2018). Only terms showing a family-wise error rate (FWER) < 0.05 were included in the analysis.

For pathway analyses, the KEGG Orthology (KO) identifiers were assigned to the significant DEGs using the blastKOALA option (https://www.kegg.jp/blastkoala/) and the pathways for responsive *Arabidopsis* root genes were visualized by the online program KEGG Mapper–Color (https://www.genome.jp/kegg/mapper/color.html). Pathways were graphically represented using the SRplot platform (Tang et al., 2023). Functional classification of DEGs was inferred using the tool MapMan v. 3.5.1.R2 (https://MapMan.gabipd.org/) (Thimm et al., 2004).

MapMan visualizations were employed for PageMan enrichment analysis. Log2fold change values of all the up-regulated and down-regulated DEGs were used as input for the PageMan analysis. Over-Representation Analysis (ORA) coupled with Fisher’s exact test was used to detect over- or underrepresented functional categories among different DEGs. A threshold of 1 was set, corresponding to at least a two-fold change in expression. Categories within the dataset that exhibited more or fewer genes than expected, and that surpassed this threshold were highlighted. The degree of deviation from the expected values was represented by varying intensities of colour. Transcription factor (TF) encoding genes were predicted using a similarity search against the PlantTFDB 5.0 database (Tian et al., 2020) with an E-value cut-off of 1E-10. The protein interaction network of *Arabidopsis* TFs was predicted by GeneMAINA (http://genemania.org/; accessed on 4th June 2024) and String (https://www.string-db.org/; medium confidence 0.400; accessed on 16th June, 2024), based on *Arabidopsis* proteins.

### 2.6 Expression analysis by qRT-PCR

The same pools of total RNA from the AsTIR19 OE-11.1 line (hereafter OE-11) and WT plants used for the construction and sequencing of the cDNA libraries (above) were used for qRT-PCR expression analysis of 15 selected stress marker genes. Genomic DNA contaminants were removed from total RNA (2 µg) by DNAse treatment, with cDNA synthesis carried out in the same tube as previously described (Morgante et al., 2013) and used as the template for qRT-PCR reactions. Specific primer pairs for *Arabidopsis* genes related to stress, defense response and hormonal pathways were designed using the Primer3 on-line tool (Untergasser et al., 2012) (**Supplementary Table S1**).

qRT-PCR reactions were conducted in three biological replicated and technical duplicates for each sample on a StepOne Plus Real-Time PCR System (Applied Biosystems, Foster City, USA), according to Morgante et al. (2013). Primer efficiencies and optimal cycle quantification (Cq) values were estimated using the online real-time PCR Miner tool (Zhao and Fernald, 2005). Transcript expression ratios were calculated and statistically analyzed using the SATqPCR web tool (http://satqpcr.sophia.inra.fr/cgi/home.cgi/, accessed in February 2024) (Rancurel et al., 2019), with normalization against *Arabidopsis* reference genes *GAPDH* and *EF-1α* (Czechowski et al., 2005).

## 3 Results

### 3.1 AsTIR19-OE lines show enhanced resistance to FOC infection in *Arabidopsis*

To assess the effects of *AsTIR19* overexpression on FOC infection, we generated five *Arabidopsis* overexpressing (OE) lines named AsTIR19-OE lines (OE1.4, OE2.6, OE6.4, OE8.2, and OE-11.1). Transformation confirmation was conducted via PCR for the marker gene BAR (Phosphinothricin N-acetyl transferase) (**Supplementary Figure S1**). Confirmation of *AsTIR19* transgene overexpression in these lines at the T2 generation was conducted via qRT-PCR, revealing slight variations in transgene expression levels among the lines (**Supplementary Figure S2**). *AsTIR19* expression was not detected in wild-type (WT) plants. Importantly, none of the OE lines exhibited discernible differences in vegetative or reproductive traits compared to WT plants, indicating that varying levels of *AsTIR19* overexpression did not lead to visible phenotypic changes in transgenic *Arabidopsis* plants.

**Figure 1:**
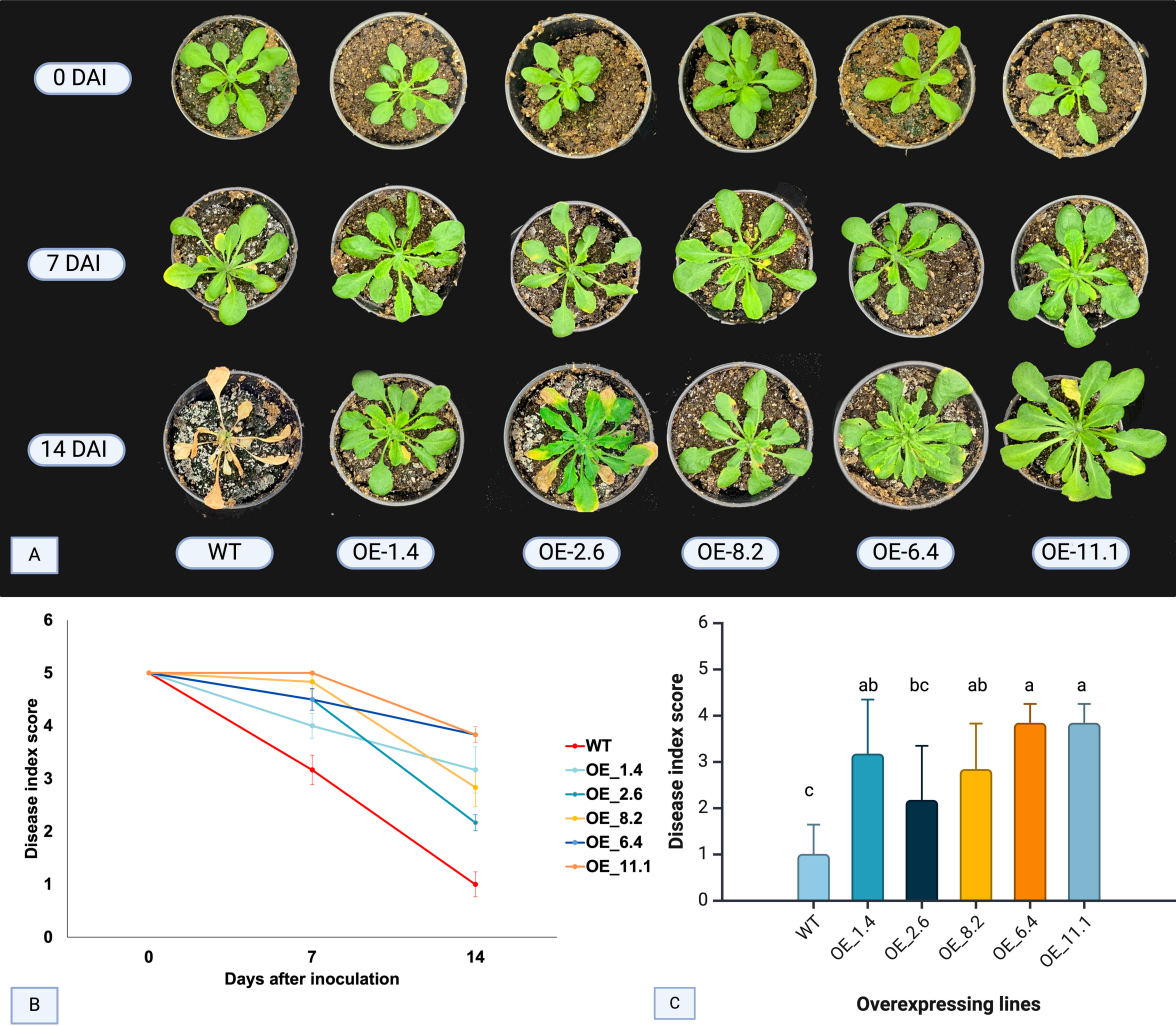
FOC disease symptoms and index score in four inoculated AsTIR19 OE lines and wild-type (WT) plants. **(A)** symptoms exhibited by AsTIR19 OE lines and WT plants at 0,7 and 14 days after FOC inoculation (DAI): **(B)** disease index score in AsTIR19 OE lines and WT plants during FOC disease progression. **(C)** disease index score in AsTIR19 OE lines and WT at 14 DAI (p<0.05); Different letters indicate a significant (p<0.05) difference in disease index score among the OE lines and WT plants.

For the FOC bioassay analysis, disease symptoms were evaluated in inoculated *Arabidopsis* OE lines and WT plants according to the scale proposed by Diener and Ausubel (2005). Initial Fusarium wilt symptoms appeared in WT plants at 7 DAI, characterized by yellowing of older leaves (**Figure 1A**). At this stage, none of the other four OE lines exhibited symptoms, except for OE-2.6 line, which showed mild symptoms. By the 14th DAI, typical FOC symptoms, such as foliar chlorosis, progressing to necrotic lesions and plant death, were prominent in most of the WT plants (**Figure 1A**). In contrast, disease progression was notably slower in all OE lines, with OE-6.4 and OE-11.1 showing no signs of disease, and OE-1.4 and OE-8.2 displaying very mild symptoms or chlorosis on older leaves (**Figure 1A**).

Assessment of FOC disease progression was conducted at 7 and 14 DAI. All five AsTIR19-OE lines showed significant differences (p<0.05) in the disease index score in comparison to WT, with 0 (zero) representing the lowest (dead plants) and 5 (five) the highest (no visible symptoms) scores (**Figure 1C**). At 7 DAI, all OE lines exhibited a higher disease index score when compared to WT plants, with increases of up to 1.6-fold. By 14 DAI, the difference between OE lines and WT plants became even more pronounced, with a maximum increase of 4-fold (**Figure 1B**). Notably, OE-11.1 and OE-6.4 displayed the highest disease indices (**Figure 1C**). Here, we showed that *AsTIR19* overexpression caused a significant reduction in FOC infection in all *Arabidopsis* OE lines tested, as demonstrated by a reduction in the disease index and a delay in the appearance of the disease symptoms such as chlorosis and leaf stunting.

To further investigate transcriptional changes underlying the increased resistance to FOC due to *AsTIR19* overexpression, we conducted a comprehensive analysis comparing the overall transcriptional behavior of AsTIR19 OE-11.1 (OE-11) line and WT plants. Additionally, we explored the impact of pathogen-challenge on both transgenic and non-transgenic WT plants. The OE-11 line was selected for transcriptional analysis due to its high disease indexes at 7 and 14 DAI, displaying a healthy phenotype without any signs of disease throughout the bioassay.

### 3.2 Transcriptome sequencing of OE-11 line and WT plants

To elucidate the molecular mechanisms underlying *AsTIR19*-modulated resistance to FOC in *Arabidopsis*, we conducted a comprehensive analysis comparing the whole transcriptome of *AsTIR19* overexpressing line (OE-11) and WT roots and their non-inoculated control counterparts. By comparing these conditions, we aimed to identify differentially expressed genes and regulatory pathways involved in the enhanced resistance conferred by *AsTIR19* in response to pathogen challenge. Overall, 12 cDNA libraries, encompassing three biological replicates of each condition, produced 152,872,554 Mb of HiSeq-4000 raw reads, of which an average of 96.3% of the total reads was successfully mapped to *A. thaliana* reference genome (version 10) (**Supplementary Table S2**). PCA (principal component analysis) analysis of the RNASeq data from the 12 cDNA libraries showed that, based on the number of counts per gene, FOC-inoculated samples (red and green) clustered together and were clearly separated from the non-inoculated samples (blue and purple), regardless of the transgene insertion (**Supplementary Figure S3**), suggesting a core infection response in OE-11 line and WT plants. Nonetheless, transgenic and WT samples each clustered together in subclusters, suggesting that, in addition to the core infection-responsive genes, specific FOC-responsive genes were regulated in the transgenic plants.

### 3.3 Transcriptome profiling changes due to AsTIR19 overexpression and FOC inoculation

**Figure 2:**
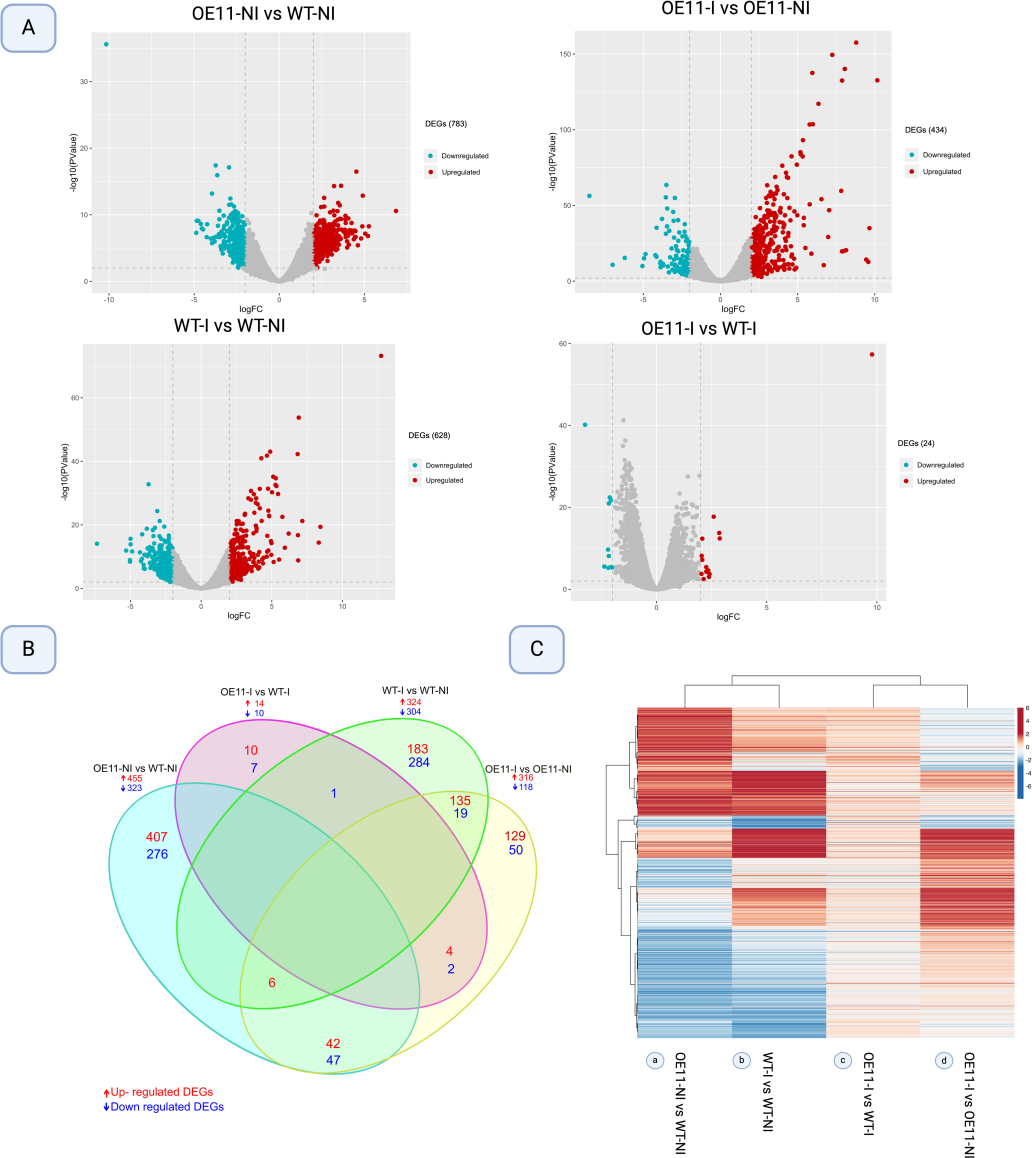
Differentially expressed genes (DEGs) (log2 Fold change >2 and P-value <0.01) in AsTIR19 OE-11 line and WT plants under FOC inoculation (I) and non-inoculation (NI) conditions. **(A)** Volcano plot analysis of differential gene expression, with −log10 (P-value) from the t-test as the y-axis and log2 (fold change) as the x-axis. Red dots represent up-regulated DEGs, and the blue dots represent down-regulated DEGs. **(B)** Venn diagram showing DEGs intersections among the four comparisons; **(C)** Heatmap plot of Hierarchical clustering distribution of DEGs in transgenic *Arabidopsis* versus WT plants FOC-inoculated and non-inoculated. Rows represent individual genes. Genes that were up- and downregulated in each comparison (columns) are indicated in red and blue, respectively.

The overall transcriptional profile of DEGs between the OE-11 line and WT plants, inoculated and non-inoculated with FOC, is represented by volcano plots in **Figure 2A**. For all comparisons, the number of upregulated DEGs (red) exceeded the number of downregulated (blue), with DEGs identified in the FOC-inoculated OE-11 line compared to non-inoculated plants (OE-11-I vs OE-11-NI) showing the highest expression magnitude (**Figure 2A**). The largest number of DEGs (778) was observed between non-inoculated OE-11 and WT plants (OE-11-NI vs WT-NI), comprising 455 up and 323 downregulated genes affected by the transgene expression (**Figure 2B**; **Supplementary Table S3**). This corresponds to approximately 2.88% of the entire *A. thaliana* genome (https://www.arabidopsis.org/).

FOC inoculation significantly altered the expression of numerous DEGs in both the OE-11 line (316 up- and 118 downregulated) and WT plants (324 up- and 304 downregulated) in comparison to non-inoculated control plants, with 179 DEGs found exclusively in the inoculated transgenic line (**Figure 2B**; **Supplementary Table S3**).

**Figure 2C** shows the overall transcriptional profile of DEGs identified between the four conditions studied. We found that the majority of DEGs due to the transgene overexpression (OE-11-NI vs WT-NI) (a) show an opposite expression behavior to those incited by FOC inoculation (OE-11-I vs OE-11-NI) in the transgenic plants (d). This overall contrasting expression behavior suggests that in *Arabidopsis*, the majority of DEGs regulated due to *AsTIR19* overexpression are not the same as those incited by FOC inoculation (**Figures 2B, C**), and different genes and metabolic pathways are triggered in response to the pathogen in the transgenic OE plants in comparison to those in the WT plants.

Manhattan plots were created to visualize the distribution of significantly expressed DEGs across the *Arabidopsis* genome, using Bonferroni correction with an FDR threshold of <0.000001 (**Supplementary Figure S4**; **Supplementary Table S4**). Using this stringent criterion, we found 29 DEGs (labelled orange dots) that are regulated due to the transgene expression (OE-11-NI vs WT-NI) (**Supplementary Figure S4A**), of which various are related to membrane receptor and signaling, such as transmembrane and amino acid transporter proteins, expansins and RLKs, as well as to reactive oxygen species (ROS) detoxification and production of defense related metabolites (**Supplementary Table S4**).

**Figure 3:**
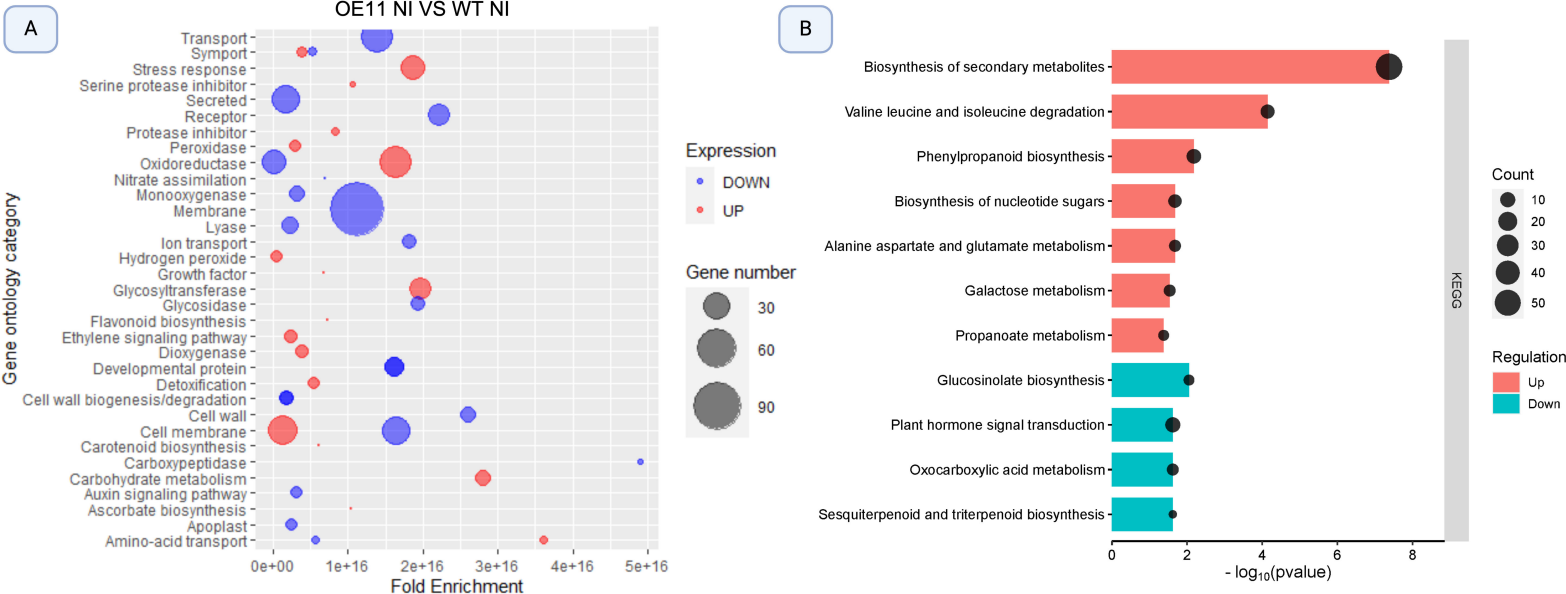
Bubble plot illustration of significantly enriched Gene Ontology (GO) terms and KEGG pathways among differentially expressed genes (DEGs) in AsTIR19 OE-11 compared to WT plants. **(A)** GO categories enriched in OE-11 line and WT plants. Bubble size correlates with Fold Enrichment (p- value < 0.05) associated with each respective GO term. Blue circles signify downregulation of DEGs, while red circles indicate upregulation; **(B)** KEGG pathways enriched in OE-11 line and WT plants. Color and size of the dots represent the regulation and the number of DEGs mapped to the indicated categories and pathways, respectively. The X-axis represents the negative logarithm of the adjusted p- value (false discovery rate - FDR) for the KEGG pathways.

From the 14 DEGs identified due to the fungal infection in the OE-11 line (OE-11-I vs OE-11-NI) (**Figure 3B**), several were associated with stress responses. These include genes involved in membrane perception (expansins) (Brasileiro et al., 2021), and genes that interfere with the accumulation of defense gene transcripts (glutamate racemase) (Kwaaitaal et al., 2011). Pathogen defense proteins identified included jacalin (Esch and Schaffrath, 2017), arginase, myrosinase (Chhajed et al., 2020) and acethyl-ornithine lectins (Kalamaki et al., 2009). Additionally, genes responsive to defense-related hormones including jasmonic (JA) and abscisic acids (ABA) were upregulated (**Supplementary Figure S4B**; **Supplementary Table S4**). Diversely, the 23 DEGs identified in the WT response to the fungal pathogen (WT-I vs WT-NI) included those related to broad stress responses, such as chitinases, wall-associated kinases (WAK) and osmotin (**Supplementary Figure S4C**; **Supplementary Table S4**). DEGs shared between both genotypes when inoculated with FOC (OE-11-I vs WT-I) included TFs such as *WRKY* and *bHLH* which have been previously associated with stress responses in *Arabidopsis* (Abe et al., 2003; Pandey and Somssich, 2009) (**Supplementary Figure S4D**; **Supplementary Table S4**).

We did not identify genomic regions or clusters of genes that were significantly differentially expressed (FDR < 0.000001) among the four conditions applied in this study, with evidenced for an even distribution of significant genes across the five *A. thaliana* chromosomes (**Figure 3**).

### 3.4 Gene ontology and KEGG pathway enrichment analysis

#### 3.4.1 DEGs due to AsTIR19 overexpression

The GO enrichment analysis revealed that upregulated DEGs (red) in the non-inoculated OE-11 line compared to WT fell into categories associated with the plant’s ability to cope with and adapt to various stresses, and signal transduction mediated by ROS (**Figure 3A**). These enriched categories included genes in “stress response”, which contained genes involved in general responses to stress; “oxidoreductase activity” which encoded enzymes that participate in oxidation-reduction reactions essential for managing oxidative stress; “ethylene signaling pathway”, which included genes crucial for various plant stress responses; “cell membrane”, that include genes potentially enhancing the structural integrity and signaling functions during pathogen attack; and “peroxidase activity”, with genes encoding peroxidases which are important for the detoxification of ROS and for strengthening cell walls. These enriched categories show a broad enhancement of stress-responsive genes in the transgenic plants overexpressing *AsTIR19* (**Figure 3A**; **Supplementary Table S3**). In addition to stress-related categories, carbohydrate metabolism was also upregulated. This is fitting because carbohydrates provide the necessary energy and resources for immune response activation (Rojas et al., 2014).

In contrast to the above, downregulated DEGs (blue) in the OE-11 line when compared to WT were enriched in categories related to “transport and secretion” encoding proteins involved in plant-microbe interactions; “membrane” including genes associated with membrane structures and possibly indicating a shift in membrane composition or function; “oxidoreductase activity” including a subset of oxidoreductases which suggests a complex regulation of redox processes, and “cell membrane and amino acid transport” including genes involved in the transport of amino acids essential for various metabolic and signaling processes (**Figure 3A**). Interestingly, the enriched categories “oxidoreductase activity” and “cell membrane” included both up- and downregulated DEGs.

The enrichment of KEGG pathways by DEGs identified in the OE-11 line when compared to WT plants revealed significant enrichment in 11 pathways, with seven pathways including up-regulated genes (**Figure 3B**). The most up-regulated enriched pathway in the OE-11 line was “biosynthesis of secondary metabolites”, which includes genes such as jacalin, chitinase, and PR proteins, that are associated with enhanced defense mechanisms, suggesting an improved defense state in the OE-11 line compared to WT plants (**Supplementary Table S3**). Another significantly enriched pathway with up-regulated genes was “valine leucine and isoleucine degradation”. The degradation of branched-chain amino acids is crucial for maintaining amino acid homeostasis and providing energy during the early phases of germination, and has been implicated in responses to abiotic stress, as noted in previous studies (Joshi et al., 2010; Gipson et al., 2017).

The upregulation of secondary metabolite biosynthesis and phenylpropanoid pathways, along with the degradation of branched-chain amino acids, indicates a complex and enhanced defense mechanism in the OE-11 line. This enhanced defense state is likely due to the ectopic expression of the *AsTIR19* gene, which appears to prime the plant for better resistance to various stresses.

#### 3.4.2 DEGs due to FOC inoculation in the OE-11 line and WT plants

**Figure 4:**
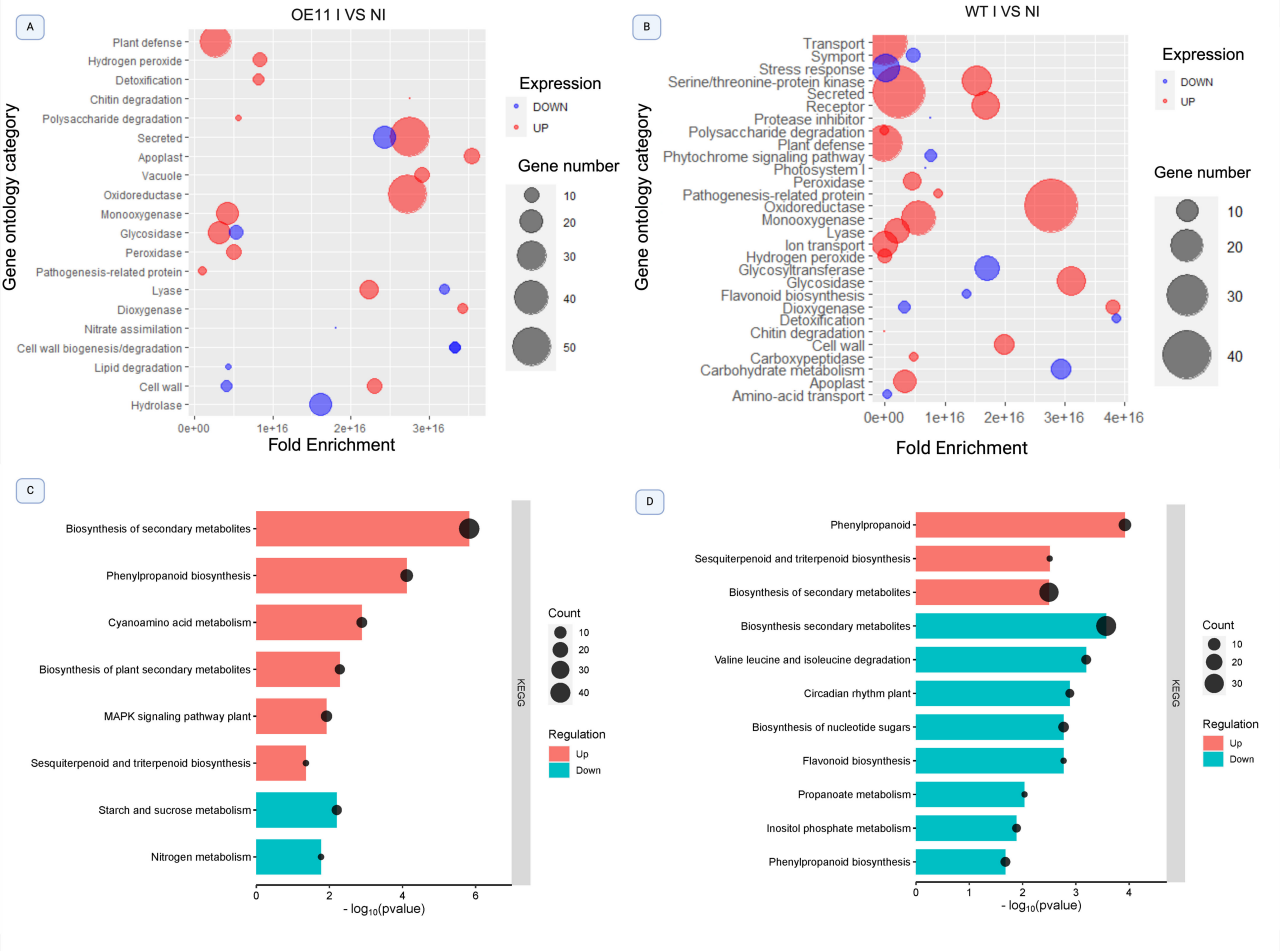
Bubble plot illustrating significantly enriched Gene Ontology (GO) terms and KEGG pathways among differentially expressed genes (DEGs) in the OE-11 line and WT plants inoculated with FOC and non-inoculated control. **(A)** GO categories enriched in the OE-11 line under FOC infection; **(B)** GO categories enriched in WT plants under FOC infection. The bubble size correlates with Fold Enrichment (p-value < 0.05) associated with each respective GO term. Blue circles signify downregulation of DEGs, while red circles indicate upregulation. **(C)** KEGG pathways enriched in the OE-11 line under FOC infection; **(D)** KEGG pathways enriched in WT plants under FOC infection. The X-axis represents the negative logarithm of the adjusted p-value (false discovery rate - FDR) for the KEGG pathways.

The most enriched GO categories in FOC-inoculated OE-11 line were “plant defense”, “secreted” and “oxidoreductase”, which contained upregulated genes with the greatest fold-changes (**Figure 4A**). Upregulated DEGs involved with “monoxygenase”, “glycosidade” and “peroxidase” activities were also enriched, suggesting the activation of defense responsive pathways and ROS response (Baxter et al., 2014). FOC-inoculated WT plants also showed an enrichment of upregulated DEGs in the categories “secreted” and “oxidoreductase” in comparison with non-inoculated (WT-I vs WT-NI) plants. However, an enrichment of downregulated DEGs in “stress response”, “glycosyltransferase”, “carbohydrate metabolism” and “flavonoid biosynthesis” categories was also observed (**Figure 4B**), all of which are functional categories involved in the plant defense response.

Most KEGG pathways enriched in the OE-11 lines after FOC inoculation consisted of upregulated DEGs involved in the production of secondary metabolites, such as “biosynthesis of plant secondary metabolites”, “phenylpropanoid biosynthesis”, and “sesquiterpenoid” and “triterpenoid biosynthesis” (**Figure 4C**). These phytochemicals are produced in response to biotic stress and often show antimicrobial activity or act as deterrents against herbivores (Guerriero et al., 2018). We also observed the enrichment of the “mitogen-activated protein kinase (MAPK)” pathway, a central metabolic pathway associated with abiotic and biotic stress adaptation, and with PAMP perception, triggering the PTI defense response (Manna et al., 2023).

In contrast, enriched KEGG pathways in the WT-inoculated plants showed a greater number of downregulated than upregulated DEGs in important biosynthetic routes linked to plant defense, such as “biosynthesis of secondary metabolites”, “flavonoid biosynthesis” and “phenylpropanoid biosynthesis”. Additionally, there was an enrichment of downregulated DEGs linked to primary metabolism pathways, such as “valine and isoleucine degradation”, “inositol phosphate metabolism”, and “biosynthesis of nucleotide sugar” (**Figure 4D**).

#### 3.4.3 Mapman analysis

We also employed MapMan analysis to depict the expression of DEGs among the four conditions studied in the context of biotic stress pathways (**Supplementary Figure S5**). An overview of the general regulation patterns of genes coding for proteins revealed upregulated DEGs in the non-inoculated OE-11 line compared to WT plants (OE-11-NI vs. WT-NI) in major biochemical pathways involved in carbohydrate and amino acid metabolism, biosynthesis of RNA, amino acids, and proteins. Conversely, genes involved in metal handling and cell wall were downregulated (**Supplementary Figure S5**). This increase in carbohydrate and protein metabolism, which was also observed in the GO enrichment analysis is likely to be attributed to the transgene overexpression and its effect on the overall metabolic activity of the plants.

Following FOC inoculation, a higher number of upregulated DEGs was observed in the OE-11 line (OE-11-I vs OE-11-NI) than in WT plants (WT-I vs. WT- NI), which are related to cell wall, stress, metal handling, and protein biosynthesis (**Supplementary Figure S5**). Additionally, genes in three categories related to biotic stress, namely “stress biotic”, “stress biotic receptors” and “stress biotic PR proteins” were upregulated only in the FOC-inoculated OE-11 line. By contrast, FOC inoculation incited downregulation of genes in the “stress biotic” category in the inoculated WT plants (WT-I vs WT-NI), but upregulation of DEGs related to cell wall, amino acids, minor carbohydrate metabolism, and metal handling (**Supplementary Figure S5**). Overall, the biotic stress categories assigned by MapMan were consistent with those enriched in the GO and KEGG analysis. This congruence underscores the reliability of the findings, indicating that *AsTIR19* overexpression reprograms the plant’s transcriptional response to better cope with FOC infection by enhancing the expression of genes involved in critical defense pathways.

### 3.5 Defense marker genes and transcription factor expression in the OE-11 line under FOC infection

#### 3.5.1 Co-expression network of transcription factors

**Figure 5:**
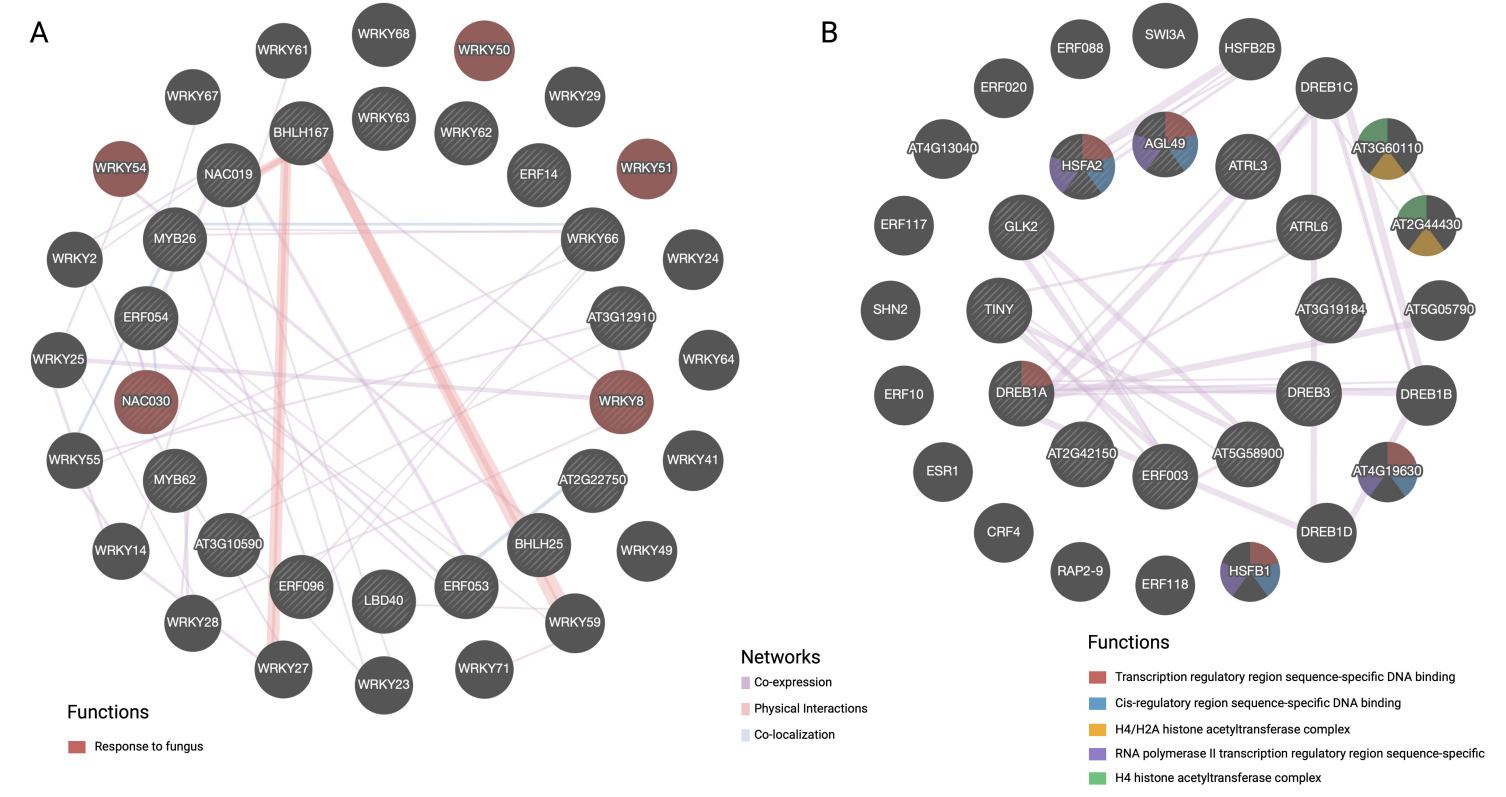
GeneMANIA analysis of transcription factors under biotic stress conditions, divided into two main networks: **(A)** Up-regulated DEGs and **(B)** Down- regulated DEGs. Nodes are colored based on their functions, as indicated by the legend. The interconnections between proteins were explored in term of physical interaction, co-expression, predicted function, co-localization, common pathway, genetic interaction, and shared protein domains.

TFs identified by PlantTFDB and GO terms enriched in the FOC-inoculated OE-11 line compared to non-inoculated plants (OE-11-I vs. OE-11-NI) were used to build protein-protein regulatory networks under biotic stress. The interconnections between proteins were explored in terms of physical interaction, co-expression, genetic interaction, and shared protein domains (**Figure 5**).

Network A illustrates upregulated DEGs coding for TFs in the OE-11 line, emphasizing specific WRKY and NAC TFs involved in responses to fungal infection (red spheres) (**Figure 5A**). While bHLH and WRKY form a substantial part of the complex regulatory networks that integrate multiple signaling pathways, cooperation between other TFs such as WRKY/MYB, bHLH/NAC and WRKY/WRKY was also observed (**Figure 5A**). These TFs are central to signaling pathways involving JA and SA, which are two major hormones in plant defense (Guo et al., 2020).

Network B showcases downregulated TFs, including those from the dehydration-responsive element-binding (DREB) family, highlighting the functional diversity of TFs in different regulatory contexts (**Figure 5B**). DREB TFs are typically more associated with abiotic stress and tend to be expressed at lower levels during biotic stress compared to bHLH, WRKY and NAC TFs (Baillo et al., 2019). The observed downregulation of drought-induced TFs from the DREB family during FOC infection suggests a contrasting regulatory pattern of genes involved in abiotic and biotic stress responses (Guo et al., 2020). TFs responsive to biotic stress such as WRKY and NAC have been integral to plant defense against fungal pathogens. Understanding their roles and interactions can help in developing strategies to enhance plant resistance to fungal diseases.

#### 3.5.2 qRT-PCR expression analysis of defense marker genes

**Figure 6:**
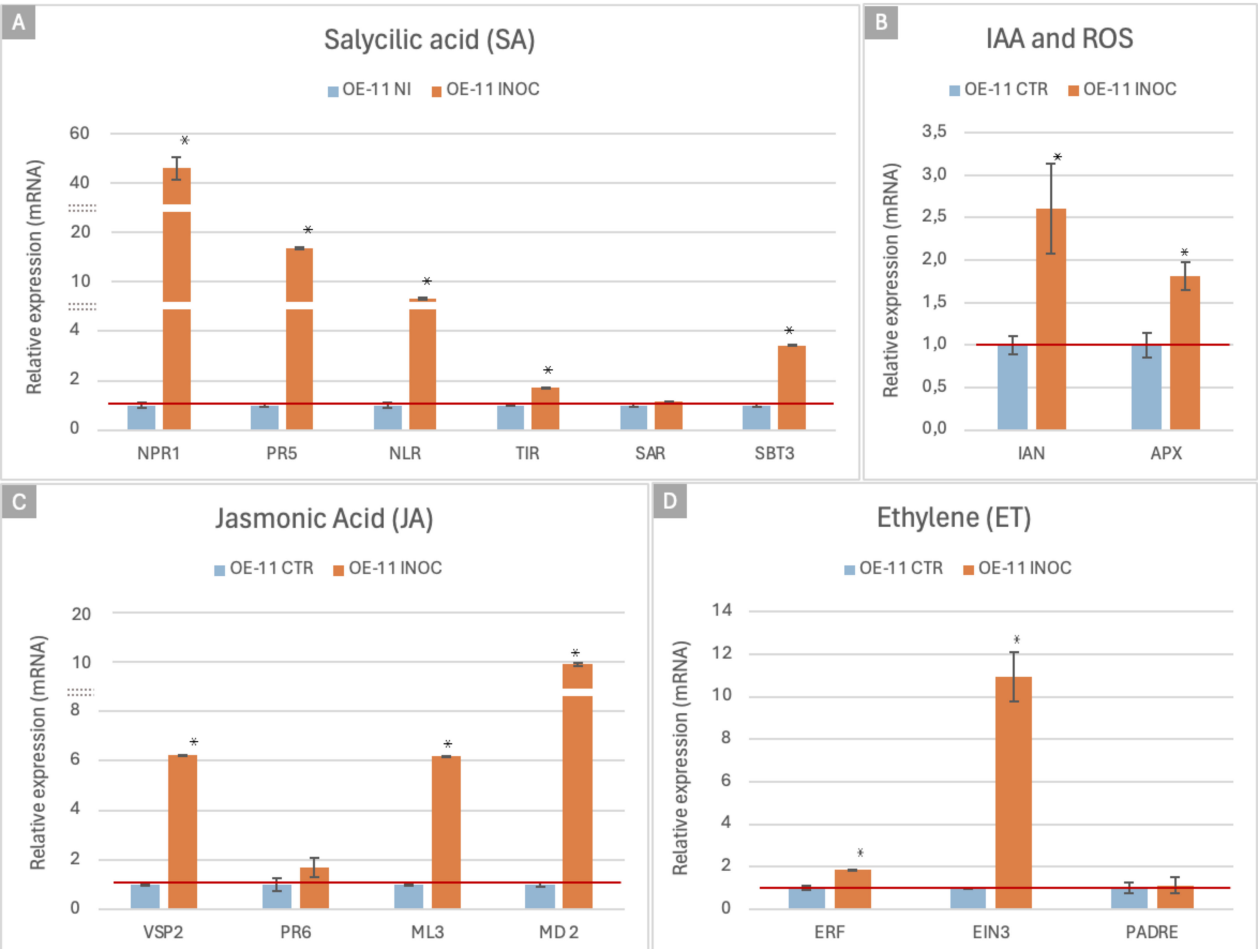
qRT-PCR expression analysis of the FOC-inoculated OE-11 line and non-inoculated plants. Relative quantification of mRNA levels of *Arabidopsis* marker genes involved in plant hormonal and defense pathways in the FOC-inoculated OE-11 line (orange) was calculated relative to WT non- inoculated plants (blue). Relative Quantification (RQ) values are calculated based on means and standard errors of nine individuals, followed by Tukey’s test (p ≤ 0.05). * indicates significant differential gene expression (p ≤ 0.05).

Plant defense responses against biotic stresses is orchestrated by signaling pathways that include salicylic acid (SA), jasmonic acid (JA) and ethylene (ET). In addition, the production of ROS is one of the first responses to pathogen attack and helps to limit pathogen spread, while IAA (indole–acetic acid) can also affect various aspects of plant defense. To investigate the role of each of these components in the FOC resistance in the OE-11 line, we analyzed the expression of 15 marker genes as indicators of the activation of specific defense pathways by qRT-PCR (**Supplementary Table S1**; **Figure 6**).

For the SA pathway, we employed marker genes involved in recognition of specific (*NLR*, *TIR*) and broadly conserved (*SBT3*) molecular pathogen features, SA signaling and regulation (*NPR1*), secondary metabolism (*PR5*), and Systemic Acquired Resistance (*SAR*). JA pathway signaling was analyzed using four marker genes, which contribute to the synthesis of defensive compounds (*VSP2*), the JA signaling pathway (*MD_2 like, ML3*) and defense proteins (*PR6*). The ET pathway marker genes included TFs (*ERF* and *EIN*) and genes involved in the recognition of pathogen derived signals (*PADRE*). Potential activation of the IAA pathway was verified using a marker gene involved in auxin signaling (*IAN*). ROS production was also analyzed by using a marker for the activation of the antioxidant system, the ascorbate peroxidase (*APX*) (**Supplementary Table S1**).

Overall, the expression of these defense markers showed a considerable increased in the FOC-inoculated OE-11 line compared to the non-inoculated plants, suggesting that the effects of the transgene overexpression are amplified by the pathogen cues (**Figure 6**). This upregulated expression trend was also observed *in silico* (RNASeq data) for all the 15 markers studied, confirming the reproducibility of our results (**Supplementary Table S3**), and suggesting that a complex network of signaling pathways was activated during the enhanced defense responses observed in the transgenic plants.

**Figure 7:**
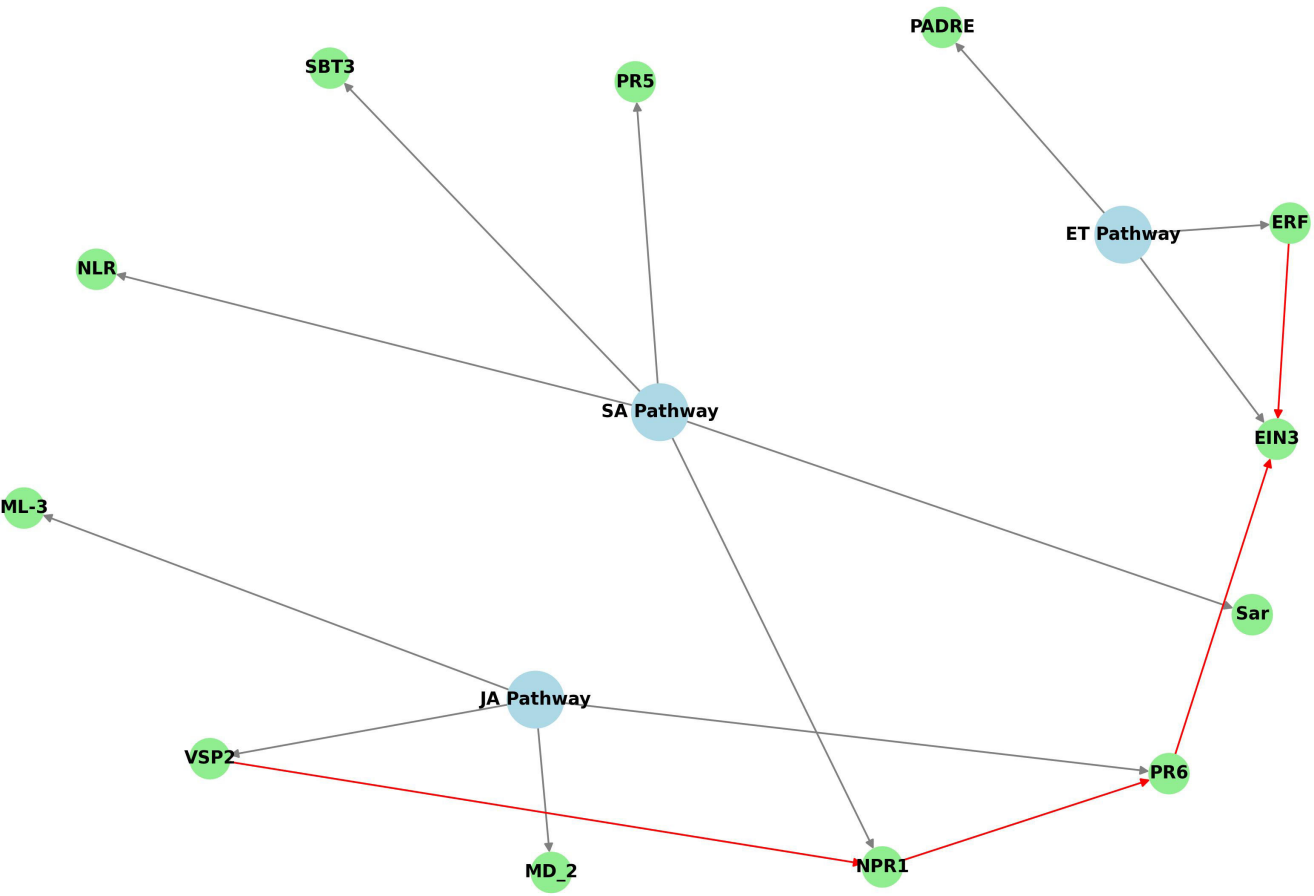
Python code-derived diagram showing the defense hormone pathways (SA, JA, ET) and their associated genes upregulated in OE-11 line inoculated with FOC. Blue nodes represent the hormonal pathways, green nodes represent the genes associated with each pathway, and grey arrows indicate the connections between genes and their respective pathways. Additional interactions are highlighted in red.

The diagram in **Figure 7** highlights key interactions among pathways involved in regulating plant defense responses. It illustrates the connections between various marker genes with upregulated expression, as determined by qRT-PCR, in the FOC-inoculated OE-11 line, together with respective hormonal signaling pathways. The JA-ET and SA signaling pathways appear to form the backbone of the plant’s immune system response in the transgenic line, while genes from the ET pathway also appear to play a modulatory role.

The relationships among the hormone signaling pathways suggest that the defense responses in the transgenic line includes various components: other NLRs (*NLR*, *TIR*), which are critical for pathogen recognition and initiation of ETI immune responses; genes linked to the recognition of pathogen-associated molecular patterns (PAMPs) and initiation of PTI response, such as *PADRE*, *SBT3* and *MD_2* and initiation of PTI response; key signaling genes from the SA pathway (*NPR1*) and JA (*VSP2*) pathways; transcription factors from the ET pathway (*ERF*, *EIN*) and genes involved in production of pathogenesis-related (PR) proteins, such as thaumatin-like protein (*PR5*) and *PR6*, which contribute to strengthening plant defenses against pathogens (**Figure 7**). In addition to these, a peroxidase gene (*APX*), which plays a crucial role in regulating ROS levels in plants, and a slightly induced SAR gene (*SAR*), were also shown to be upregulated in the OE-11 line.

The pathway interactions observed in the OE-11 line reveal a multi-layered defense mechanism that include signaling and *crosstalk* among the three hormones that primarily regulate plant defense against biotic stress: SA, JA and ET (**Figure 7**). We suggest that the overexpression of *AsTIR19* activates SA signaling, directly promoting the upregulation of defense-related genes (*PR5*), while also modulating the ET and JA pathways via *NPR1*, which activates genes in the JA pathway (*VSP2* and *PR6*) (**Figure 7**). This coordination not only enhances resistance and the production of antimicrobial compounds, but also contributes to a more sustained defense mechanism through systemic acquired resistance (SAR), contributing to the OE-11 line’s enhanced ability to resist FOC infection.

## 4 Discussion

The extensive diversity of host-specific forms within *F. oxysporum*, known as *formae speciales*, is likely due to the predominant mode of asexual reproduction and the gene-for-gene interactions with specific host plants (Katan and Di Primo, 1999; Michielse and Rep, 2009). Given that many affected plant species are in tropical and subtropical regions, the incidence of the pathogen could potentially increase due to global warming (Berrocal-Lobo and Molina, 2008).

*F. oxysporum* produces effectors that suppress or evade primary and secondary plant immune responses, and cell wall-degrading enzymes that are required for superficial root colonization and penetration, playing an important role during pathogenesis (Wang et al., 2022b). Previous studies have shown that, in addition to be functional in different plant-pathogen systems (Staal et al., 2008; Son et al., 2021), truncated NLRs can perform regulatory roles in tolerance responses to abiotic stresses, act as “helpers” for other NLRs, and condition broad-spectrum resistance (Tamborski and Krasileva, 2020). This was supported by our previous studies, in which the overexpression of a truncated NLR gene (*AsTIR19)* isolated from the fungal-resistant *A. stenosperma* (Michelotto et al., 2015; De Macedo Leal-Bertioli et al., 2010) led to a significant reduction in infection caused by the necrotrophic fungus *S. sclerotiorum* (Guimaraes et al., 2022).

In this study, we generated five *Arabidopsis* AsTIR19-OE lines and analyzed the effects of this truncated NLR on the innate immune response against FOC. As the pathogen colonizes vascular tissues, the degree of wilt symptoms, chlorosis and vascular colonization all correlate with the success in restricting vascular colonization (Gao et al., 1995; Diener and Ausubel, 2005). Here, all OE lines showed fewer visible symptoms of chlorosis and wilt, resulting in a significantly higher index score (up to 4 times) compared to WT plants at 14 DAI, demonstrating a strong effect of the transgene on reducing FOC infection in *Arabidopsis*. As previously observed for other truncated NLRs (Zhao et al., 2015; Barragan et al., 2021; Son et al., 2021) and specifically for *AsTIR19* against necrotrophic fungi (Guimaraes et al., 2022), where overexpression enhanced resistance to *S. sclerotiorum*, we suggest that this NLR plays a role in the recognition of diverse effectors from hemibiotrophic and necrotrophic fungal pathogens, and amplification of the plant immune response.

Overall, an upregulation of genes was observed in OE lines in comparison to WT, inoculated and non-inoculated with FOC. However, the interaction between plants and pathogenic fungi can also result in the downregulation of specific host defense genes, facilitating resource allocation, stress management, and modulation of immune responses (Nishad et al., 2020). Pathogens are known to employ various strategies to down-regulate host genes, including the secretion of effector proteins that inhibit defense-related genes, manipulation of hormone signaling pathways, particularly involving salicylic acid, jasmonic acid and ethylene, and the induction of negative regulators of plant defense (Patkar and Naqvi, 2017; Han and Kahmann, 2019). At the specific timepoint investigated in this study (7 DAI), we observed contrasting expression patterns in some defense-related genes against FOC in *Arabidopsis* OE lines challenged and non-challenged with the pathogen. These included NLRs, leucine-rich proteins, and receptors for microbe-associated molecular patterns (MAMPs) and pathogen-associated molecular patterns (PAMPs), including receptor-like kinases (RLKs) and receptor-like proteins (RLPs). Additionally, we noted changes in expression of genes encoding the cell wall-related proteins pectin lyases and expansins, as well as secondary metabolites such as concanavalin and thioredoxin, alongside critical transcription factors (TFs) such as MYB protein (RSM1), ERF, and DREB. While these genes were upregulated in the transgenic OE line when compared to non-inoculated transgenic plants, they were downregulated during pathogen infection when compared to non-inoculated transgenic plants. These findings highlight the intricate interplay between plant defense mechanisms and pathogen strategies in transgenic plants, with the downregulation of defense-related genes during pathogen infection underscoring the challenges to enhance plant resistance to pathogens through targeted genetic and biotechnological approaches.

Previous studies in *Arabidopsis* have shown that the perception of *F. oxysporum* by plants follows the concept of elicitor-induced immune response, which in turn activates several plant defense-signaling pathways, such as those mediated by the plant hormones SA, ET, JA and ABA (Berrocal-Lobo and Molina, 2008; Zhu et al., 2013; Chen et al., 2014; Wang et al., 2022a). Here, whole transcriptome sequencing of the transgenic OE-11 line revealed that DEGs induced by the overexpression of *AsTIR19* differed significantly from those triggered by FOC infection. Specifically, DEGs upregulated by *AsTIR19* overexpression were predominantly linked to Gene Ontology (GO) categories associated with pathogen perception, such as cell membrane proteins (expansins, pectate lyases, transmembrane proteins) and cell wall modifying enzymes (glycosyltransferases), ROS (peroxidases, methyltransferases) and carbohydrate metabolism (galactosyltransferases). In contrast, after fungal infection, DEGs associated with secondary defense compounds, particularly those involved in phenylpropanoid and terpenoid biosynthesis (lectins, racemases and myrosinases), ROS-related enzymes (peroxidases and methyltransferases), and PR proteins were more commonly upregulated. These results suggest that *AsTIR19* overexpression enhances plant resilience to FOC infection, potentially through modulation of both stress response pathways and specific defense mechanisms.

### 4.1 AsTIR19 overexpression activated multiple plant defense responses

In a more detailed comparative analysis of the OE-11 line and WT transcriptomes, we found that the effects of the overexpression of this truncated NLR led to an enrichment of functional GO categories linked to general stress defense mechanisms, such as stress response and oxidoreductase activity. The global transcriptional reprogramming observed in the AsTIR-OE line mirrors that triggered by endogenous plant NLRs, which, after being activated by receptors that recognize the pathogen, increase ROS production, defense hormone production, and immune signaling (Lolle et al., 2020). Moreover, the enrichment in the carbohydrate metabolic process observed in the transgenic plants is also crucial in plant defense against pathogens. Carbohydrates provide the necessary energy and resources for immune response activation and regulate the expression of PR proteins in response to pathogens (Rojas et al., 2014).

The overexpression of *AsTIR19* also significantly affects cellular metabolic pathways, immune response mechanisms, and specialized biosynthetic processes. Accordingly, upregulated DEGs in the enriched KEGG pathways are related to phenylpropanoid biosynthesis, including flavonoids, monolignols, phenolic acids, stilbenes, and coumarins. These compounds are essential components of cell walls, protectants against high light and UV radiation, and phytoalexins against herbivores and pathogens (Deng and Lu, 2017). We also observed the enrichment in the biosynthesis of secondary metabolites category in the transgenic plants, including propanoates. These are known precursors of propionic acid, which has been demonstrated to inhibit the mycelial growth of various fungi, including *B. cinerea, S. sclerotiorum* and *F. oxysporum* (Şehirli and Saydam, 2016).

In addition to triggering defense genes and associated pathways, the overexpression of *AsTRI19* also induced several genes associated with primary metabolic pathways in *Arabidopsis*, such as those involved in the synthesis or degradation of carbohydrates, amino acids, and lipids. Sugars constitute a primary substrate providing energy and structural material for plant defense responses in plants and can also act as signal molecules interacting with hormonal signaling networks in plant tissues (Morkunas and Ratajczak, 2014). Here, an increase of upregulated DEGs associated with alanine, aspartate, and glutamate metabolism, as well as those involved in the degradation of the valine, leucine, and isoleucine was also observed in transgenic *AsTIR19* plants. This is in accordance with studies in *Arabidopsis* showing that higher levels of sugars and accumulation of amino acids in pathogen-infected plant tissues can lead to enhanced plant resistance (Rojas et al., 2014; Ward et al., 2010).

### 4.2 Transcriptome analysis of AsTIR19 OE plants in response to FOC inoculation

The transcriptome analysis of transgenic *AsTIR19*-OE plants in response to FOC inoculation revealed a significant increase in the number of upregulated DEGs in GO categories related to plant defense responses. This included chitinases, thaumatin-like pathogenesis-related proteins (PRs), and peroxidase genes previously associated with the early stages of the plant defense response to SA treatment in *Arabidopsis* (Agrawal et al., 2010). This suggests that the transgenic plants are activating a comprehensive defense response involving both direct antimicrobial actions and signaling pathways. Such reprogrammed transcriptional networks induced by biotic and abiotic stresses in transgenic plants has previously been observed in *Arabidopsis* overexpressing heterologous Transcription Factors (TFs), in poplar overexpressing multiple resistance genes, and in transgenic wheat overexpressing *GmDREB* for enhanced tolerance to drought and salt (Quan et al., 2019; Fu et al., 2021; Zhang et al., 2014; Jiang et al., 2014).

Chitinases and thaumatin-like proteins are PR proteins involved in both direct and indirect defense reactions (Dong et al., 2017; Wang et al., 1996). While chitinases play a dual role, both inhibiting pathogen growth, and releasing Pathogen-Associated Molecular Patterns (PAMPs) that induce defense response in the host (Zhang et al., 2016), thaumatin-like proteins (PR5) are involved in systemically acquired resistance (SAR) and stress responses in plants. In addition, accumulation of ROS and the activation of plant defense enzymes, such as peroxidases, helps to maintain cell integrity and remove accumulated peroxide. The higher expression of PR and peroxidase coding genes in the FOC-inoculated compared to non-inoculated OE plants, suggests that the cues from the pathogen trigger a more robust defense response in OE plants.

Accordingly, the enrichment of KEGG pathways related to the biosynthesis of secondary and phenylpropanoid metabolites, including the mitogen-activated protein kinase (MAPK) signaling pathways, was also highly enriched in the FOC-inoculated transgenic line. MAPK cascades are activated by a variety of stress stimuli leading to changes in gene expression and hormone responses (Romeis et al., 1999; (Taj et al., 2010). The functional analysis of DEGs between FOC-inoculated and non-inoculated OE line suggests that fungal infection can trigger defense responses mediated by SA and JA pathways through MAPK signaling. This activation subsequently incites the production of ROS and their scavenging enzymes, as well as the expression of defense genes (Jagodzik et al., 2018).

It is important to note that many of the rapid changes in gene expression observed during the OE-line responses to FOC infection also occur in the susceptible interaction. Accordingly, DEGs identified between FOC-inoculated and non-inoculated WT plants were also found in stress-responsive GO categories, such as plant defense and secreted proteins, although with a smaller fold enrichment than those observed in the transgenic line. In contrast, while KEGG pathway enrichment analysis revealed that upregulated DEGs in biosynthesis of secondary metabolites pathways, including flavonoid biosynthesis and propanoate metabolism, were enriched in the transgenic line, downregulated DEGs were observed in these categories in the WT plants.

Overall, the comparative functional analysis between the FOC-inoculated transgenic line and WT plants highlight the effects of *AsTIR19* overexpression on the induction of defense pathways linked to the biosynthesis of secondary metabolites and phenylpropanoids. These pathways are further enriched by genes involved in biosynthesis of terpenoids, phenolic compounds, and cyano amino acids, which likely lead to an enhanced disease resistance in the transgenic line (Häusler et al., 2014; Zaynab et al., 2018).

### 4.3 AsTIR19 overexpression induced TF activation

TFs are integral to plant defense mechanisms, primarily through their roles in regulating secondary metabolism, activating defense-related genes, and interacting with hormonal signaling pathways. Their cooperative interactions are crucial for mounting an effective defense response against a range of stresses (Buscaill and Rivas, 2014; Wani et al., 2021).

Here, we found that TFs from four families that play an important role in immune responses, such as bHLH, WRKY, NAC and MYB were significantly upregulated in the FOC-inoculated transgenic line in comparison to the control. bHLH TFs are involved in the activation of genes related to stress responses, including those encoding antimicrobial peptides and enzymes, such as those involved in the regulation of JA pathways and biosynthesis of flavonoids and anthocyanin (Li, 2014; Naik et al., 2022). WRKY proteins interact with other TFs and regulatory proteins to modulate crucial biological processes, helping to perceive external stress stimuli through signaling molecules. These TFs are also involved in the SA pathway and the activation of primary genes associated with stress responses, including specific defense responses to *F. oxysporum* (Jiang et al., 2017; Aamir et al., 2018; Li et al., 2021). In this study, we found that bHLH and WRKY TFs form part of a complex regulatory network that integrate multiple signaling pathways in the AsTIR19-OE plants. This network has the potential to modulate defense responses to pathogens. Specifically, WRKY TFs, which are involved in SA pathway, can interact with bHLH TFs that regulate the JA pathway. Such interactions enable a coordinated modulation of defense mechanisms, potentially enhancing the plant’s ability to respond effectively to diverse pathogenic threats.

Additionally, NAC TFs, which are known to play a functional role in regulating responses to multiple stresses (Nuruzzaman et al., 2013; Saidi et al., 2022; Srivastava et al., 2022), were upregulated in the OE-line. The overexpression of a *NAC* gene in wheat led to significant transcriptional reprogramming in response to *F. graminearum*, potentially enhancing defenses primarily involved in the hormone-mediated signaling pathways of JA and abscisic acid (ABA) (Vranić et al., 2023).

The cooperative interaction among TFs observed in AsTIR-OE plants during interaction with FOC also seems to contribute to the resistance in the transgenic plants against the pathogen. This has previously been demonstrated by studies that highlight protein-protein interactions between TFs regulating the expression of specific PR genes in response to disease and contributing to SA and JA-induced pathogen resistance (Tran et al., 2007; Shan et al., 2016; Nuruzzaman et al., 2013).

### 4.4 AsTIR19 overexpression upregulated plant defense marker genes and hormonal pathways

Marker genes associated with specific biological pathways have been frequently used as indicators for the positive/negative regulation of their respective signaling cascade (Zhang et al., 2020b). In our study, we observed via qRT-PCR a significant upregulation in the expression of a subset of 15 commonly used defense marker genes in the transgenic line, following FOC inoculation in comparison to non-inoculated plants. Overall, the FOC-inoculated OE-line exhibits a complex and multi-faceted defense response. This is characterized by the upregulation of various marker genes in the SA signaling pathway, indicating a robust SA-dependent defense mechanism, in concurrency with upregulated genes in the JA-ET pathway, indicating a coordinated defense response. Additionally, the upregulation of marker genes in the IAA hormone pathway and ROS scavenging suggests their additional role in modulating the defense response. These findings collectively highlight a complex and integrated defense mechanism activated in the OE-line against FOC infection.

By acting as a central regulator of the SA-mediated defense pathway, NPR1 ensures a robust and effective immune response by activating the transcription of PR genes (Spoel et al., 2009; Cao et al., 1997). Here, the strong upregulation of *NPR1* and *PR5* (fold change>40 and >16 respectively) in inoculated transgenic plants underscores the importance of the SA pathway in providing strong defense against FOC, corroborating the potential benefits of genetic modifications that enhance endogenous *NPR1* expression for improving disease resistance in transgenic plants.

In addition to the increased production of PR proteins, two previously studied *Arabidopsis* NLR genes, *NLR* (At1g72910) and the truncated *TIR* (At1g72930) (Nasim et al., 2020; Chen et al., 2021), were also upregulated after FOC infection in the transgenic plants. This suggests that *AsTIR19* may work in concert with such other NLRs as recently shown by Ngou et al. (2022). In these cooperative networks, beyond the role of individual NLRs, an intricate receptor network requires multiple NLRs to function together for recognizing diverse pathogen effectors and triggering immune signaling. However, further research is required to elucidate the interaction between *AsTIR19* and these NLRs, and to clarify their molecular relationships.

The subtilase *SBT3* gene was also upregulated in the FOC-inoculated transgenic line. *SBT3* is known to contribute to the activation of defense-related pectin methylesterases (PMEs), influencing the expression of specific defense genes and the structure of pectin (Coculo et al., 2023). Its expression initiates a durable auto-induction mechanism that promotes chromatin remodeling and activates a SA-dependent mechanism of priming of defense genes for amplified response (Ramírez et al., 2013).

Activation of the JA pathway in the transgenic line during interaction with FOC was denoted by the upregulation of two genes involved in pathogen perception, namely *MD-2-like* and *ML3*. These genes are implicated in immune responses to pathogenic cell components, such as lipopolysaccharides and other PAMPs, as well as to molecules associated with herbivory (HAMPS), that can trigger immune responses via JA –mediated pathways (Fridborg et al., 2013; Song et al., 2022). Additionally, JA signaling and production of antimicrobial compounds is demonstrated by the upregulation of *VSP2.* This gene product contributes to the plant’s ability to resist pathogens by enhancing the production of secondary metabolites, other defense-related compounds and PR6, with their associated antimicrobial properties contributing to either death or inhibition of pathogens, including fungi and bacteria (Myagmarjav et al., 2017).

As previously observed in tobacco plants infected by *S. sclerotiorum* (Guimaraes et al., 2022), and in our current study with *A. thaliana* infected by *F. oxysporum*, overexpression of the NLR *AsTIR19* driven by a constitutive promoter led to an ubiquitous transgene expression, with the activation of common primary SA and JA-ET defense pathways in these two pathosystems which involved necrotrophic and hemibiotrophic fungal pathogens and two distinct plant host species.

In addition to SA and JA signaling pathways, the ethylene pathway was triggered in the FOC-inoculated OE line, as demonstrated by the upregulation of the TFs *EIN3* and *ERF*, which are central to mediation of the plant’s response to ethylene (Zhao et al., 2021). Additionally, a member of the PADRE gene family, which is involved in the regulation of pathogen-induced stress responses (Didelon et al., 2020), was also slightly upregulated in the transgenic FOC-inoculated OE line.

In parallel with the primary regulated defense hormones pathways described above, we also observed a potential enhancement of the auxin signaling pathway and ROS signaling, as evidenced by the upregulation of *IAN* and *APX* genes, respectively. Auxin signaling can modulate various defense responses, including the activation of PR genes and the reinforcement of the plant cell wall (Remans et al., 2006). This hormone can also influence the plant’s overall defensive capacity by regulating growth and developmental processes in response to stress. ROS are crucial plant defense molecules that serve multiple functions. They are known to act as signaling molecules that trigger defense-related genes and proteins, exert direct antimicrobial actions on microorganisms, contribute to cell wall reinforcement through oxidative cross-linking, and are involved in programmed cell death to restrict pathogen spread (Ali et al., 2018). Together, the triggering of these various defense pathways in the AsTIR-OE line highlights a complex network of plant defense mechanisms that work synergistically to enhance resistance in the transgenic plant against FOC.

Persistence of Fusarium wilt disease can be attributed to the complex genetics of host resistance, constituting a difficult trait to confer by breeding, together with the persistence of the pathogen in the field for extended periods (Gordon, 2017). Enhanced resistance against *Fusarium* wilt disease in different plant species has been achieved with variable success through the engineering of host genes involved in resistant interactions and defense responses (Hao et al., 2020; Zhang et al., 2020a; Chitwood-Brown et al., 2021; Sadhu et al., 2023), and through host-induced gene silencing (HIGS) via RNAi (Singh et al., 2020). In this study, we demonstrated that the overexpression of *AsTIR19*, a truncated NLR from wild *A. stenosperma*, enhanced tolerance to FOC in transgenic *Arabidopsis*. Transcriptome profiling revealed that the transgene overexpression alone affected certain defense genes and regulators, and metabolic pathways, affected by the transgene overexpression. We also identified genes and their related regulation networks that were altered in transgenic plants in response to FOC. These results thus contribute to elucidating the mechanisms by which, after fungal effector recognition, the amplification of the immune response and the regulation of key genes occurs due to *AsTIR19* overexpression.

*AsTIR19* is, therefore, a potential candidate for plant improvement through biotechnology approaches and constitutes a promising tool for resistance against FOC. Moreover, our previous studies showed that *AsTIR19* overexpression in transgenic tobacco also enhanced resistance against the necrotrophic pathogen *S. sclerotiorum* (Guimaraes et al., 2022). Together, these findings suggested that a broader resistance towards plant pathogenic fungi may be achieved by overexpressing this truncated NLR gene in different host plants. *AsTRI19* gene, therefore, has the potential to be explored in a range of susceptible crops, and plant breeding programs can profit through the development of tightly linked molecular markers, enabling precise selection and introgression of this transgene into elite cultivars. Additionally, the pyramiding of *AsTIR19* with other resistance genes offers a robust strategy to manage pathogen variability and reduce the likelihood of resistance breakdown over time. Such a multi-gene approach can ensure sustained protection against a broad spectrum of *F. oxysporum* strains, contributing to more resilient crop varieties and improved agricultural productivity.

## Data Availability

The data presented in the study are deposited in the SRA database of NCBI repository, accession number PRJNA1125443.

## Supplementary material

Supplementary Figure 1 PCR amplification patterns from ten *Arabidopsis thaliana* OE-lines. PCR amplification patterns in *Arabidopsis thaliana* DNA samples using primers specific to the *bar* gene (amplicon size 405 bp). Lanes 2 to 11: DNA from the respective *A. thaliana* OE-lines at T1 generation: 1.4; 2.6; 6.4; 7.9; 8.2; 9.5; 10.4; 11.1; 13.5 and 15.6. Lane 12: +CTR = DNA from wild-type (WT) non-transgenic *A. thaliana* plants mixed with the binary vector pPZP−AsTIR19 (positive control); Lane 13: -CTR = No template DNA control (negative control); Lane 14: WT = DNA from wild-type (WT) non-transgenic *A. thaliana* plants (negative control). Ladder = 1 Kb Plus DNA Ladder (Catalog # 10787026; Thermo Fisher Scientific Inc.).

Supplementary Figure 2 Expression levels of *AsTIR19* relative to the two reference genes in overexpressing (OE) lines of *Arabidopsis thaliana* (OE- 1.4, OE-11.1, OE- 2.6, OE- 6.4, OE- 8.2).

Supplementary Figure 3 PCA analysis of RNASeq data from OE-11 line and WT plants.

Supplementary Figure 4 Manhattan plot representations displaying the position and frequency of the differentially expressed genes (DEGs) over the five *A. thaliana* chromosomes. **(A)** DEGs between non-inoculated OE-11 line and WT plants; **(B)** DEGs between FOC-inoculated and non-inoculated OE-11 line; **(C)** DEGs between FOC-inoculated and non-inoculated WT plants; **(D)** DEGs between FOC-inoculated OE-11 line and WT plants. The x-axis is divided into the five chromosomes, and genes are plotted in their respective positions. The y-axis represents the expression values, calculated as -10*log (p value). Dotted red lines indicate the threshold for false discovery rate (FDR < 0.000001). Orange dots represent DEGs, with the TAIR ID of those with higher -log10 (p-values) highlighted.

Supplementary Figure 5 Pageman-derived overview of molecular functional categories in DEGs observed in OE-11 and WT plants inoculated with FOC and non-inoculated plants. Upregulated genes are indicated by red squares and downregulated by blue squares. Statistical significances are represented by a false color heat map (up- red; down- blue).
