# Supplementary figures and images for: Ectopic expression of a truncated NLR gene from wild Arachis enhances resistance to Fusarium oxysporum
Source: Front Plant Sci. 2024 Nov 13;15:1486820. doi: 10.3389/fpls.2024.1486820 (PMC11598430; doi:10.3389/fpls.2024.1486820)

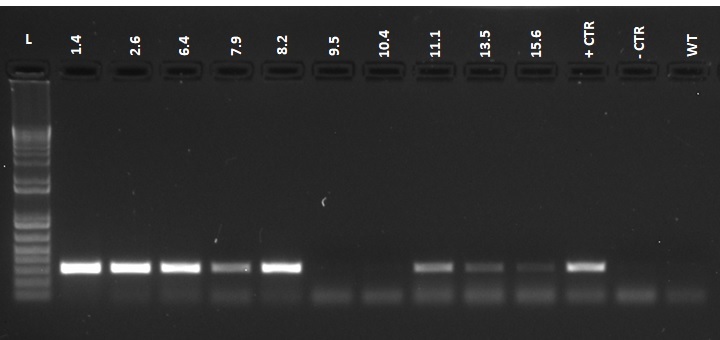

Supplement: Supplementary Figure 1 — PCR amplification patterns from ten Arabidopsis thaliana OE-lines. PCR amplification patterns in Arabidopsis thaliana DNA samples using primers specific to the bar gene (amplicon size 405 bp). Lanes 2 to 11: DNA from the respective A. thaliana OE-lines at T1 generation: 1.4; 2.6; 6.4; 7.9; 8.2; 9.5; 10.4; 11.1; 13.5 and 15.6. Lane 12: +CTR = DNA from wild-type (WT) non-transgenic A. thaliana plants mixed with the binary vector pPZP−AsTIR19 (positive control); Lane 13: -CTR = No template DNA control (negative control); Lane 14: WT = DNA from wild-type (WT) non-transgenic A. thaliana plants (negative control). Ladder = 1 Kb Plus DNA Ladder (Catalog # 10787026; Thermo Fisher Scientific Inc.). [file Image1.jpeg]

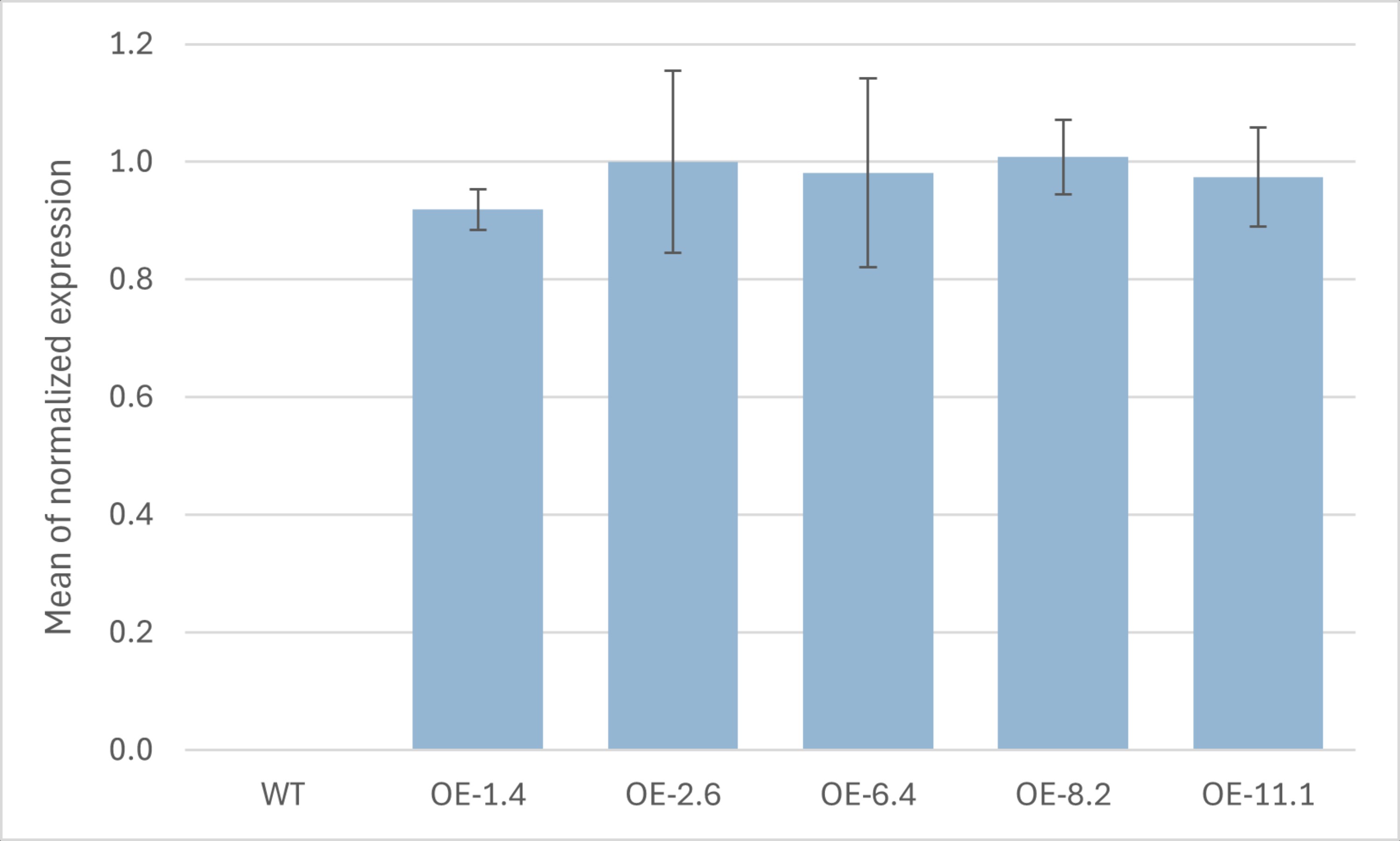

Supplement: Supplementary Figure 2 — Expression levels of AsTIR19 relative to the two reference genes in overexpressing (OE) lines of Arabidopsis thaliana (OE- 1.4, OE-11.1, OE- 2.6, OE- 6.4, OE- 8.2). [file Image2.jpeg]

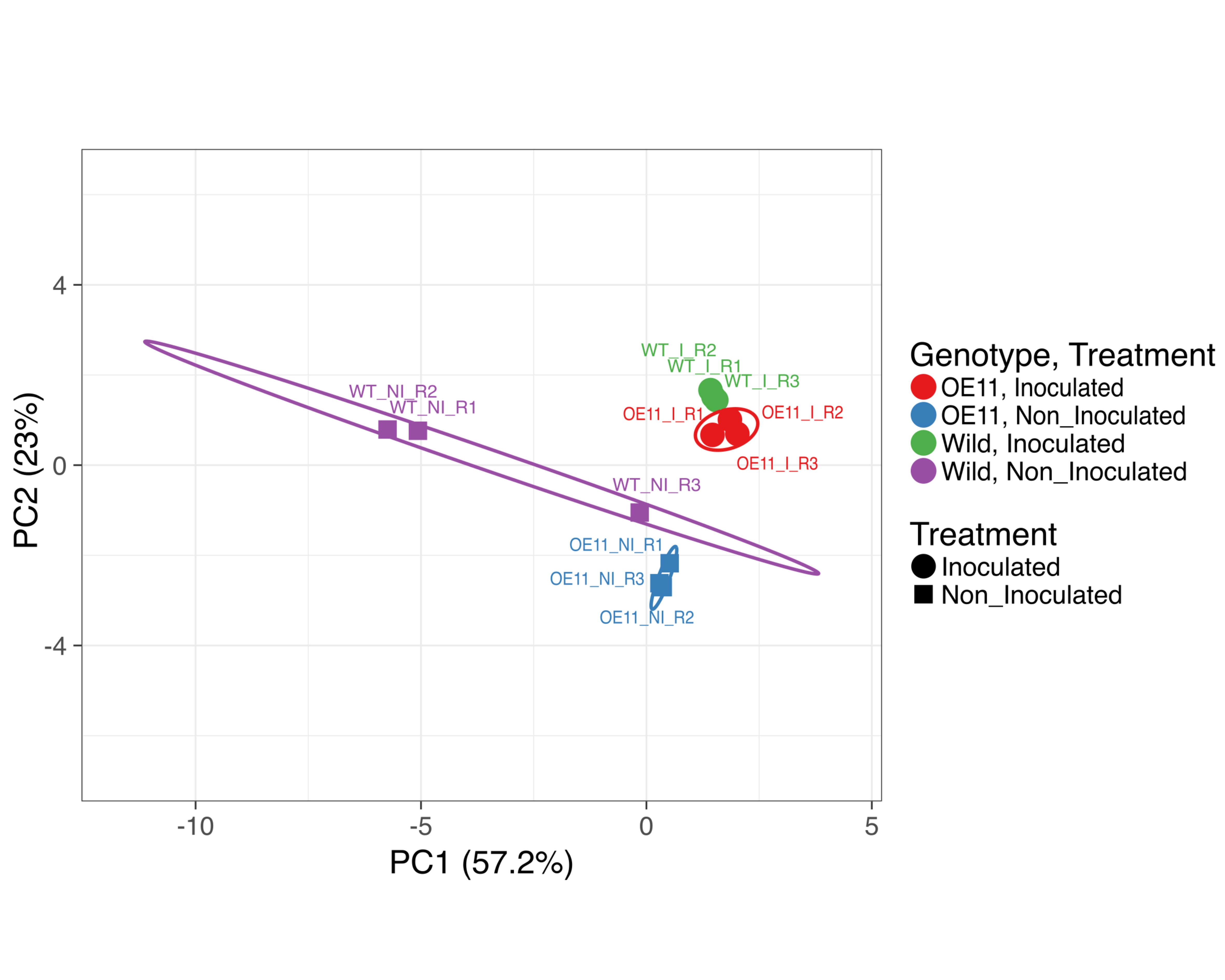

Supplement: Supplementary Figure 3 — PCA analysis of RNASeq data from OE-11 line and WT plants. [file Image3.jpeg]

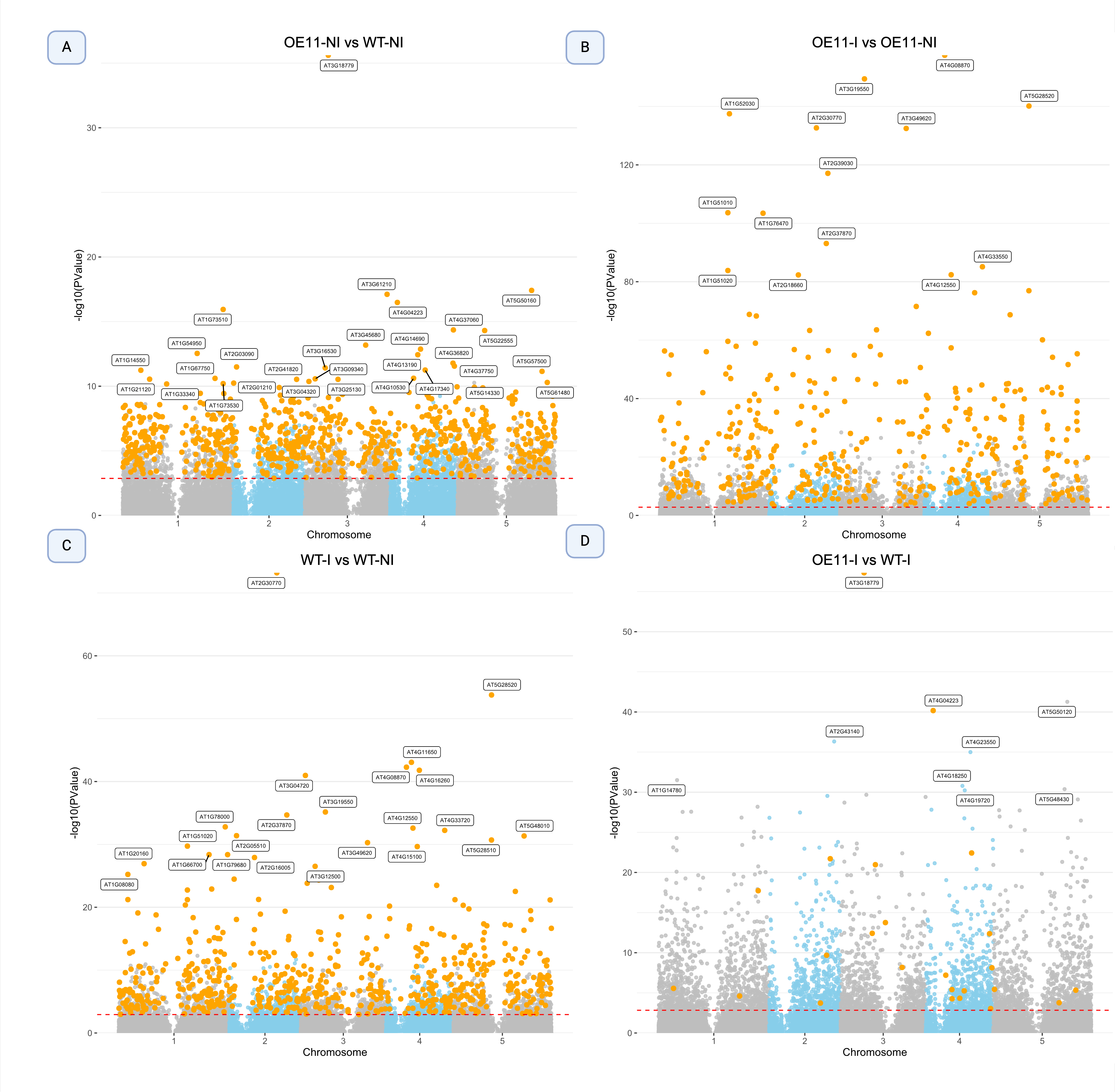

Supplement: Supplementary Figure 4 — Manhattan plot representations displaying the position and frequency of the differentially expressed genes (DEGs) over the five A. thaliana chromosomes. (A) DEGs between non-inoculated OE-11 line and WT plants; (B) DEGs between FOC-inoculated and non-inoculated OE-11 line; (C) DEGs between FOC-inoculated and non-inoculated WT plants; (D) DEGs between FOC-inoculated OE-11 line and WT plants. The x-axis is divided into the five chromosomes, and genes are plotted in their respective positions. The y-axis represents the expression values, calculated as -10*log (p value). Dotted red lines indicate the threshold for false discovery rate (FDR < 0.000001). Orange dots represent DEGs, with the TAIR ID of those with higher -log10 (p-values) highlighted. [file Image4.jpeg]

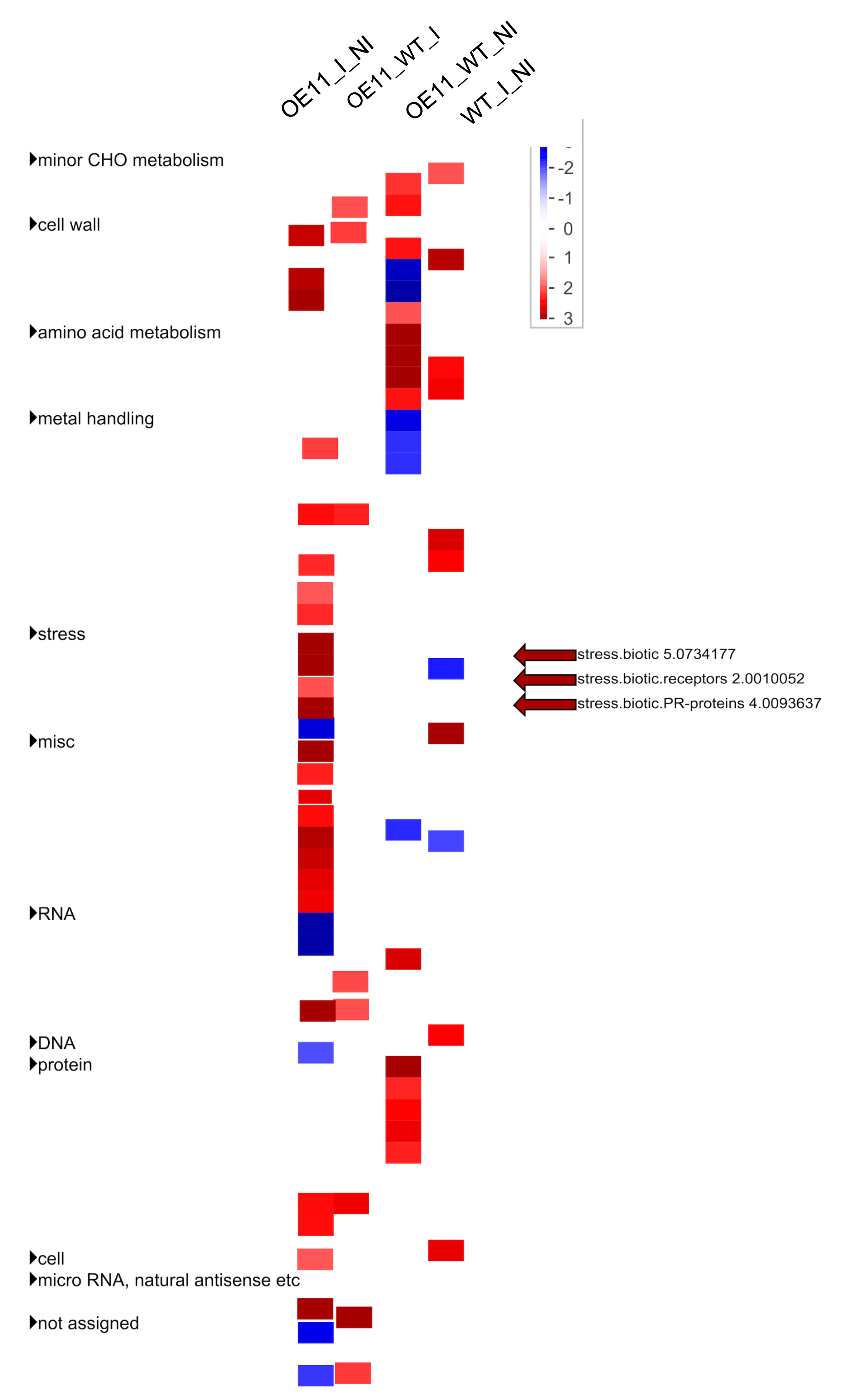

Supplement: Supplementary Figure 5 — Pageman-derived overview of molecular functional categories in DEGs observed in OE-11 and WT plants inoculated with FOC and non-inoculated plants. Upregulated genes are indicated by red squares and downregulated by blue squares. Statistical significances are represented by a false color heat map (up- red; down- blue). [file Image5.jpeg]
